# A machine-learning-based prediction of non-home discharge among acute heart failure patients

**DOI:** 10.1007/s00392-023-02209-0

**Published:** 2023-05-03

**Authors:** Akira Okada, Hidehiro Kaneko, Masaaki Konishi, Kentaro Kamiya, Tadafumi Sugimoto, Satoshi Matsuoka, Isao Yokota, Yuta Suzuki, Satoko Yamaguchi, Hidetaka Itoh, Katsuhito Fujiu, Nobuaki Michihata, Taisuke Jo, Hiroki Matsui, Kiyohide Fushimi, Norifumi Takeda, Hiroyuki Morita, Hideo Yasunaga, Issei Komuro

**Affiliations:** 1 Department of Prevention of Diabetes and Lifestyle-Related Diseases, Graduate School of Medicine, The University of Tokyo, Tokyo, Japan; 2 The Department of Cardiovascular Medicine, The University of Tokyo Hospital, 7-3-1, Hongo, Bunkyo-Ku, Tokyo, 113-8655 Japan; 3 The Department of Advanced Cardiology, The University of Tokyo, Tokyo, Japan; 4 Department of Medical Science and Cardiorenal Medicine, Yokohama City University Graduate School of Medicine, Kanagawa, Japan; 5 Department of Rehabilitation, School of Allied Health Sciences, Kitasato University, Kanagawa, Japan; 6 Department of Cardiology and Nephrology, Mie University Graduate School of Medicine, Tsu, Japan; 7 Department of Biostatistics, Hokkaido University Graduate School of Medicine, Sapporo, Japan; 8 Center for Outcomes Research and Economic Evaluation for Health, National Institute of Public Health, Saitama, Japan; 9 The Department of Health Services Research, The University of Tokyo, Tokyo, Japan; 10 Department of Clinical Epidemiology and Health Economics, The University of Tokyo, Tokyo, Japan; 11 Department of Health Policy and Informatics, Tokyo Medical and Dental University, Tokyo, Japan

**Keywords:** Heart failure, Machine learning, Non-home discharge, Clinical epidemiology, Claims database analysis

## Abstract

**Background:**

Scarce data on factors related to discharge disposition in patients hospitalized for acute heart failure (AHF) were available, and we sought to develop a parsimonious and simple predictive model for non-home discharge via machine learning.

**Methods:**

This observational cohort study using a Japanese national database included 128,068 patients admitted from home for AHF between April 2014 and March 2018. The candidate predictors for non-home discharge were patient demographics, comorbidities, and treatment performed within 2 days after hospital admission. We used 80% of the population to develop a model using all 26 candidate variables and using the variable selected by 1 standard-error rule of Lasso regression, which enhances interpretability, and 20% to validate the predictive ability.

**Results:**

We analyzed 128,068 patients, and 22,330 patients were not discharged to home; 7,879 underwent in-hospital death and 14,451 were transferred to other facilities. The machine-learning-based model consisted of 11 predictors, showing a discrimination ability comparable to that using all the 26 variables (*c*-statistic: 0.760 [95% confidence interval, 0.752–0.767] vs. 0.761 [95% confidence interval, 0.753–0.769]). The common 1SE-selected variables identified throughout all analyses were low scores in activities of daily living, advanced age, absence of hypertension, impaired consciousness, failure to initiate enteral alimentation within 2 days and low body weight.

**Conclusions:**

The developed machine learning model using 11 predictors had a good predictive ability to identify patients at high risk for non-home discharge. Our findings would contribute to the effective care coordination in this era when HF is rapidly increasing in prevalence.

**Supplementary Information:**

The online version contains supplementary material available at 10.1007/s00392-023-02209-0.

## Introduction

The number of patients with heart failure (HF) is increasing worldwide. Currently, there estimated to be 64 million HF patients globally [1]. It is considered a critical epidemiological condition globally [2–4], and a serious issue in Asia as well [5–7]. Due to the super-aging society, the prevalence of HF is rapidly increasing in Japan. It is estimated that there are approximately 1 million HF patients in Japan, and the number of patients with HF is predicted to increase continuously, reaching 1.3 million by 2030 [8]. The Japanese Circulation Society conducted the Japanese Registry Of All cardiac and vascular Diseases (JROAD) to collect the data from almost all teaching hospitals with cardiovascular beds. According to JROAD, the annual number of patients with acute HF (AHF) hospitalization increased by 22% from 212,793 in 2012 to 260,157 in 2016 (while that of acute myocardial infarction increased by 6% during the same period) [9]. To overcome this critical condition due to increasing AHF, preparing the optimal medical care system which enables seamless treatments (acute- and chronic-phase) for patients with AHF is essential [10]. From this point of view, it is essential to integrate the comprehensive care system for HF patients and to develop a medical coordination system in a community. For this end, it would be clinically valuable to predict the discharge disposition of hospitalized patients owing to AHF. Generally, the main goal of health care providers is to discharge hospitalized AHF patients home. However, some patients have difficulty being discharged home, and we must find a suitable facility to which each patient would be transferred accordingly in a community. This process is burdensome for not only health care providers but also patients and their families. If discharge disposition can be predicted in the early stages of hospitalization, we will be able to simultaneously proceed with acute care for patients with AHF and the process of medical coordination in a community, thereby efficient acute management, smooth healthcare coordination, shorter hospital stays, and ultimately, lower inpatient care costs could be achieved. Nevertheless, clinical evidence on discharge disposition and its determinants has been scarce. Here, we aimed to identify determinants of discharge to other facilities (not to home) using a nationwide inpatient database. We believe that it is important to overview the state of management for AHF focusing on discharge disposition using large-scale data at this timing when the critical epidemiological condition owing to increase in the number of AHF patients is rapidly approaching worldwide.

## Methods

### Data source

This study is a retrospective cohort study using data extracted from the Japanese Diagnosis Procedure Combination (DPC) database, a Japanese national inpatient database. The details of the DPC database have been explained previously [11, 12]. The DPC database has detailed information on admitted patients. The following data on patient information were available: age; sex; body height and weight; activities of daily living based on the Barthel Index; consciousness level at time of admission; admission status (scheduled/unscheduled and use of ambulance service); several diagnosis names (main diagnosis, diagnosis requiring admission, and comorbidities existing at the time of admission recorded as *International Classification of Diseases, 10*^*th*^* Revision* (ICD-10) codes and text data in Japanese; daily procedures performed or daily drugs administered during hospitalization. The DPC database has had information as to the origin of patients among those discharged since April 2014, i.e., whether the patient was transferred from another hospital or nursing facility or not.

We had the study protocol on this study approved by the institutional review board of the University of Tokyo [approval number: 3501-(3)]. Because we used data that had been anonymized, the necessity for informed consent was waived.

### Study design and population

Using the DPC database, we extracted data on patients undergoing an unscheduled admission, not transferred from nursey facilities or other hospitals, for the treatment of AHF of New York Heart Association (NYHA) classification ≥ II on record from April 1, 2014 to March 31, 2018. The ICD-10 codes used to identify AHF were I50.0, I50.1, and I50.9, and these diagnoses were required to be recorded as a diagnosis requiring admission and the main diagnosis of admission. The exclusion criteria were as follows: missing information on discharge disposition; age < 20 years; length of hospital stay < 3 days; and patients with missing data on Barthel index or body mass index (BMI).

#### Variables and candidate predictors

We used the following data from the extracted dataset: age, sex, BMI, smoking status, Japan Coma Scale score at admission, Barthel Index, the severity of AHF at admission, comorbidities at admission, use of ambulance, and several kinds of procedures performed within 2 days of hospital admission.

Age, BMI, and Barthel index were treated as continuous variables. Consciousness level at admission was dichotomized into two groups, based on whether the patient had impaired consciousness or not. The severity of HF was based on NYHA classification (Class II-IV) as categorical variables. We used comorbidities present at admission based on the ICD-10 codes as follows: anemia (ICD-10 codes starting with D46, D5, D60-64); hypertension (ICD-10 codes starting with I10-13, I15); ischemic heart disease (ICD-10 codes starting with I21-24); diabetes (ICD-10 codes starting with E10-14); chronic pulmonary disease (ICD-10 codes starting with I27, J40-45, J60–J62, J64-67, and J70); chronic liver disease (ICD-10 codes starting with B18, K70-74, and K76); kidney failure (ICD-10 code starting with N18 or N19); cancer (ICD-10 codes starting with C or D0); and osteoporosis (ICD-10 code starting with M800-805, M808, M809, M810-816, M818, M819, M820, M821, or M828). We also considered cardiac complications present at admission based on the ICD-10 codes as follows: shock status (ICD-10 codes starting with R57); ventricular tachycardia/fibrillation (ICD-10 codes starting with I490); and dilated cardiomyopathy (ICD-10 codes starting with I42). Among diagnoses present at admission except AHF, we also used the weighted total of comorbidities using Charlson comorbidity index as defined [13]; therefore, the value of 0 can exist. We prepared a binary variable showing whether the patient had hospitalized within the prior 30 days. Use of following drugs or procedures within 2 days were also used as binary variables: loop diuretics; vasopressors; intensive care unit; enteral alimentation initiation; kidney replacement therapy (intermittent dialysis or continuous hemodiafiltration) and intensive cardiopulmonary support (tracheal intubation, intra-aortic balloon pumping, or extracorporeal membrane oxygenation).

Candidate predictive factors were selected among those used on previous studies [11, 12, 14] and variables that are clinically relevant and can be obtained in the DPC database. Specifically, the candidate risk factors included patient age; sex; BMI; Barthel index on admission; consciousness level on admission; the severity of HF based on NYHA criteria; comorbidities present on admission such as hypertension, ischemic heart disease, diabetes mellitus, pulmonary disease, liver disease, dilated cardiomyopathy, kidney failure or kidney replacement therapy, shock status or ventricular tachycardia/fibrillation, osteoporosis, and anemia or receipt of transfusion; Charlson comorbidity index; day of the week at admission; procedures generally reflecting the severity of AHF. We also included the following variables that were clinically effective in treating patients: whether to initiate enteral alimentation, rehabilitation within 2 days of admission. Furthermore, we added the following binary variables associated with severity or comorbidity: whether to undergo use of intensive cardiopulmonary support, loop diuretics, intravenous vasopressors, or intensive care unit within 2 days of admission.

#### Study outcomes and variables

The primary outcome was not going home after treatment for AHF. In the primary analysis, we included in-hospital death in the outcome. We performed an analysis stratified by age, sex, and Barthel index. The cutoff of age and Barthel index category division, was 80 years and 60, respectively, either of which was the median value among the whole population.

### Statistical analysis

#### Patient characteristics

We summarized the patients’ demographics based on the site of discharge. Patient characteristics were compared across the places of discharge using the chi-squared test or the Wilcoxon rank-sum test.

#### Identifying risk factors and developing a prediction model

We classified the included patients into a training set consisting of randomly selected 80% and a test set using the remaining 20% of the total patients.

To create a model that has both a better discrimination ability and a clinical usability, we used binomial logistic models with L1-penalized estimation (least absolute shrinkage and selection operator, Lasso regression). Considering the trade-off between explainability versus complexity among machine learning methods, the Lasso regression focuses on explainability and facilitates interpretation of the prepared model by performing variable selection via shrinkage of the coefficients of some noninformative variables to 0 [15]. Regarding Lasso regression, we used one standard error (1SE) rule to achieve more parsimonious models; 1-SE rule of Lasso regression was described in details and used to make parsimonious models recently [16]. Briefly, ordinary Lasso regression is a machine learning that uses the optimal value for the hyperparameter *λ* to maximize the frequency of correct categorization with tenfold inner cross-validation, and 1SE rule utilizes the maximal *λ* within 1SE of the difference. Candidate variables are all available variables that are considered important in this study of AHF.

We compared two models in terms of discrimination ability and calibration ability: one using 1SE-selected variables and the other using all the 26 variables.

#### Model validation

We evaluated the model in terms of discriminative ability and calibration in the test set (the remaining 20% of the sample). Regarding discriminative ability evaluation, we measured the predictive performance using c-statistics (area under the receiver operating characteristic curve). The c-statistic was described with its 95% confidence intervals (CI). We showed c-statistic in each selected variable and its stepwise change based on the variable importance prepared by the machine learning. To assess the calibration of the prepared model, we used “*pmcalplot*” in *Stata* to describe from the calibration curve the following components: calibration in the large (CITL) index, showing the difference between the average predicted probabilities and the observed event frequencies, whose ideal value should be zero; (ii) the calibration slope, whose ideal value should be one; and (iii) the expected probability vs observed frequency (E:O) ratio, whose ideal value should be one as used in several previous studies [17–19].

#### Sensitivity analyses

We conducted three sensitivity analyses for the primary outcome. First, we prepared the predictive model after changing the time window for observing procedures or drug administration from the first 2 days to the first 3 days of hospitalization to confirm that the window period (2 days) was not arbitrary and that the gained information was robust. Next, as another sensitivity analysis, we regarded patients with length of stay ≥ 30 days as patients undergoing non-home discharge, because hospital stay this long may have been due to the inability of the family members or caregivers to bring the patient home smoothly, which may equal to non-home discharge. Finally, we excluded those undergoing in-hospital death, and performed the analysis, because discharge dead may differ in character from non-home discharge alive. We performed all statistical analyses by use of Stata Version 17 (StataCorp, College Station, TX, USA), and used a two-tailed significance level of *P* < 0.05. Part of the Stata script used in this study is stored as Supplementary Fig. 1.

##### Patient and public involvement statement

Patients were not involved in the design, or conduct, or reporting, or dissemination plans of our research.

## Results

### Study population

**Fig. 1:**
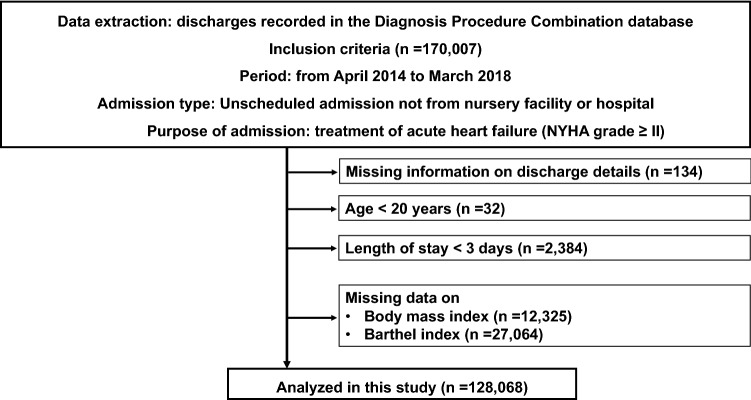
Flowchart of patient selection. *DPC* Diagnosis procedure combination

We present the patient selection flowchart as Fig. 1. Among all the eligible patients in the DPC database from April 2014 to March 2018, we identified 170,007 patients undergoing an unscheduled admission for treatment of AHF with NYHA grade ≥ II; a total of 41,939 patients were excluded (Fig. 1).

The 128,068 eligible patients hospitalized for AHF in 817 hospitals were analyzed. Overall, 22,330 (17.4%) patients underwent in-hospital death (*n* = 7,879) or non-home discharge, that is, transferred to other facilities (*n* = 14,451) such as nursery home, after treatment of AHF (Table 1). Those who could not go home after treatment for AHF were more likely to be older, female, lean, have consciousness disturbance, more severe AHF, history of readmission for heart failure within previous 30 days, such comorbidities as kidney failure, ischemic heart disease, or cancer, and undergo such procedures as transfusion for anemia or intensive therapy related to AHF treatment.

**Table 1:** Characteristics of eligible patients

Variable	Category	Home discharge	Non-home discharge or in-hospital death	*p*-value
		*n* = 105,738	*n* = 22,330	
Age		80.0 (71.0–86.0)	85.0 (79.0–89.0)	< 0.001
Age category	< 65 years	14,894 (14.1%)	1,240 (5.6%)	< 0.001
	65–74 years	21,146 (20.0%)	2,425 (10.9%)	
	75–84 years	38,081 (36.0%)	7,202 (32.3%)	
	85– years	31,617 (29.9%)	11,463 (51.3%)	
Male		60,349 (57.1%)	10,880 (48.7%)	< 0.001
Body mass index (kg/m^2^)		22.5 (20.0–25.4)	21.1 (18.7–23.8)	< 0.001
Body mass index category	–18.49 kg/m^2^	14,443 (13.7%)	5,297 (23.7%)	< 0.001
	18.5–24.99 kg/m^2^	62,040 (58.7%)	13,076 (58.6%)	
	25.00 kg/m^2^	29,255 (27.7%)	3,957 (17.7%)	
Barthel index		70 (25–100)	20 (0–65)	< 0.001
Consciousness on admission	Alert	94,315 (89.2%)	16,376 (73.3%)	< 0.001
	Impaired	11,423 (10.8%)	5,954 (26.7%)	
Categorization based on New York Heart Association	Class II	31,310 (29.6%)	4,193 (18.8%)	< 0.001
	Class III	41,561 (39.3%)	7,860 (35.2%)	
	Class IV	32,867 (31.1%)	10,277 (46.0%)	
Admission on weekends or holidays		22,821 (21.6%)	5,480 (24.5%)	< 0.001
With readmission history for heart failure within previous 30 days		7,481 (7.1%)	1,834 (8.2%)	< 0.001
Hypertension		61,613 (58.3%)	9,341 (41.8%)	< 0.001
Diabetes mellitus		31,215 (29.5%)	5,424 (24.3%)	< 0.001
Kidney failure or kidney replacement therapy receipt within 2 days		13,233 (12.5%)	3,907 (17.5%)	< 0.001
Liver disease		2,435 (2.3%)	499 (2.2%)	0.54
Pulmonary disease		8,056 (7.6%)	1,601 (7.2%)	0.021
Ischemic heart disease		1,231 (1.2%)	495 (2.2%)	< 0.001
Cancer		4,752 (4.5%)	1,143 (5.1%)	< 0.001
Dilated cardiomyopathy		5,256 (5.0%)	809 (3.6%)	< 0.001
Shock or ventricular fibrillation on admission		1,028 (1.0%)	558 (2.5%)	< 0.001
Osteoporosis		2,009 (1.9%)	356 (1.6%)	0.002
Charlson comorbidity index		0.0 (0.0–1.0)	0.0 (0.0–2.0)	< 0.001
Anemia on admission or transfusion within 2 days		8,490 (8.0%)	2,345 (10.5%)	< 0.001
Intensive cardiopulmonary support within 2 days		14,756 (14.0%)	4,208 (18.8%)	< 0.001
Intravenous loop diuretics within 2 days		73,336 (69.4%)	15,932 (71.3%)	< 0.001
Intravenous vasopressors within 2 days		14,810 (14.0%)	5,141 (23.0%)	< 0.001
Intensive care unit use within 2 days		17,420 (16.5%)	4,640 (20.8%)	< 0.001
Enteral alimentation within 2 days		95,161 (90.0%)	17,523 (78.5%)	< 0.001
Rehabilitation within 2 days		12,251 (11.6%)	2,533 (11.3%)	0.30

Data are presented as median (interquartile range) for continuous measures, and *n* (%) for categorical measures

### Determination of factors related to the failure to return home alive after hospitalization

**Fig. 2:**
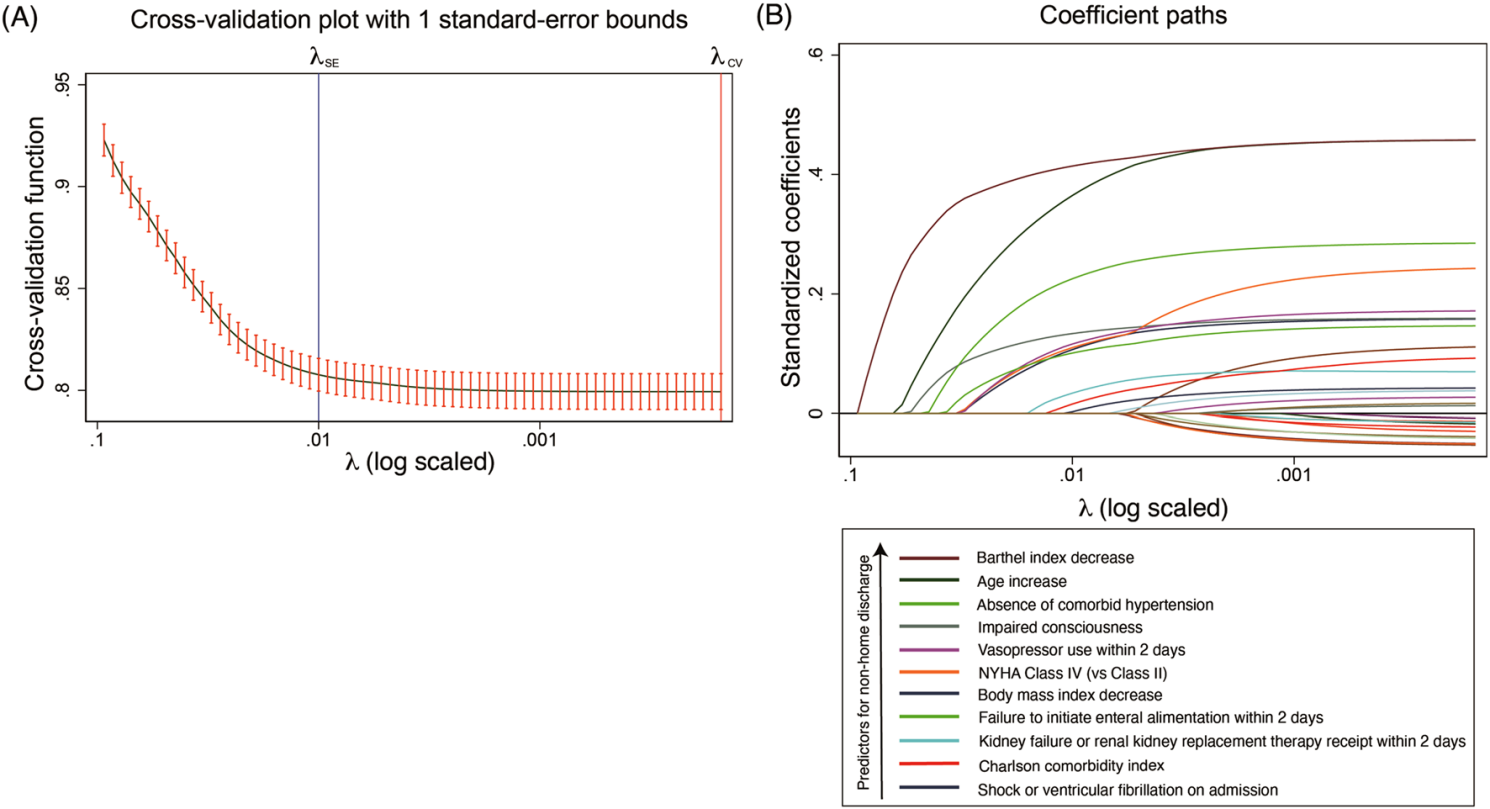
Cross-validation plot and coefficient paths of variable selection. (**A**) Cross-validation plot of mean squared error corresponding to smoothing parameter λ with standard errors. (**B**) Coefficient paths of variable selection. Legends show the selected predictors selected at the point of λ_SE_ in the order of standardized coefficient values. *λ*_SE_, the largest *λ* among *λ* for which the cross-validation function is within one standard error of the minimum of the cross-validation function (*λ* = 0.0100); *λ*_CV_, *λ* where the cross-validation function is minimum (*λ* = 0.000152); *NYHA* New York Heart Association

**Fig. 3:**
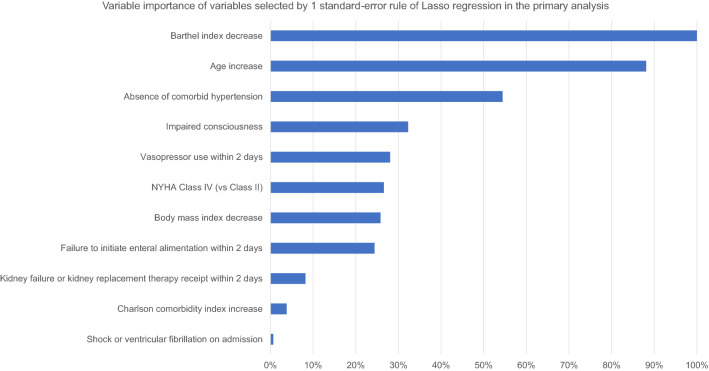
Variable importance of the variables selected by 1 standard-error rule of lasso regression. *SE* standard-error, *NYHA* New York Heart Association

We first divided the whole population into the training set (80%) and the test set (20%) (Supplementary Table 1). The process of variable selection using the 1SE rule of Lasso regression is shown in Fig. 2. We selected the value of hyperparameter *λ* in the following fashion: after determining *λ*_CV_, where the cross-validation function resulted in the minimized value, we found *λ*_SE_, which is the largest *λ* while being within the 1SE of the lowest mean squared error. Thus, from the 1SE rule of Lasso regression, we constructed the model using variables selected by Lasso regression (model using 1SE-selected variables) to predict non-home discharge, and with use of this *λ*_SE_, 11 variables were selected, and the variable importance generated via machine learning is shown in Fig. 2B. The most important factor for the prediction of the non-home discharge was a lower Barthel index on admission (odds ratio, 1.12 per 10 points decrease; 95% CI 1.11–1.12), followed by higher age (odds ratio, 1.49 per 10-year increase; 95% CI 1.46–1.52), the absence of comorbid hypertension, the impaired consciousness on admission, the use of vasopressors, the highest NYHA class (Fig. 3). The coefficients of regression using the variables selected by the 1SE rule is shown in Table 2. In the model using all the 26 variables, the coefficients of the multivariable regression are shown in Supplementary Table 2.

**Table 2:** Odds ratios of the variables selected by 1 standard-error rule of Lasso regression in predicting non-home discharge or in-hospital death

Variable	Category	Odds ratio	95% confidence interval	*P* value
Age (10-year increase)		1.49	1.46–1.52	< 0.001
Body mass index (5-kg/m^2^ decrease)		1.20	1.18–1.23	< 0.001
Barthel index (10-point decrease)		1.12	1.11–1.12	< 0.001
Consciousness on admission	Alert	Reference		
	Impaired	1.58	1.51–1.65	< 0.001
Categorization based on New York Heart Association	Class II	Reference		
	Class III	1.26	1.20–1.32	< 0.001
	Class IV	1.63	1.56–1.71	< 0.001
Absence of comorbid hypertension		1.79	1.73–1.86	< 0.001
Kidney failure or kidney replacement therapy receipt within 2 days		1.28	1.22–1.34	< 0.001
Charlson comorbidity index		1.07	1.05–1.08	< 0.001
Shock or ventricular fibrillation on admission		1.50	1.32–1.71	< 0.001
Intravenous vasopressors within 2 days		1.57	1.50–1.64	< 0.001
Failure to initiate enteral alimentation within 2 days		1.52	1.45–1.60	< 0.001

### Model validation in terms of predictive ability and calibration of the developed models

**Fig. 4:**
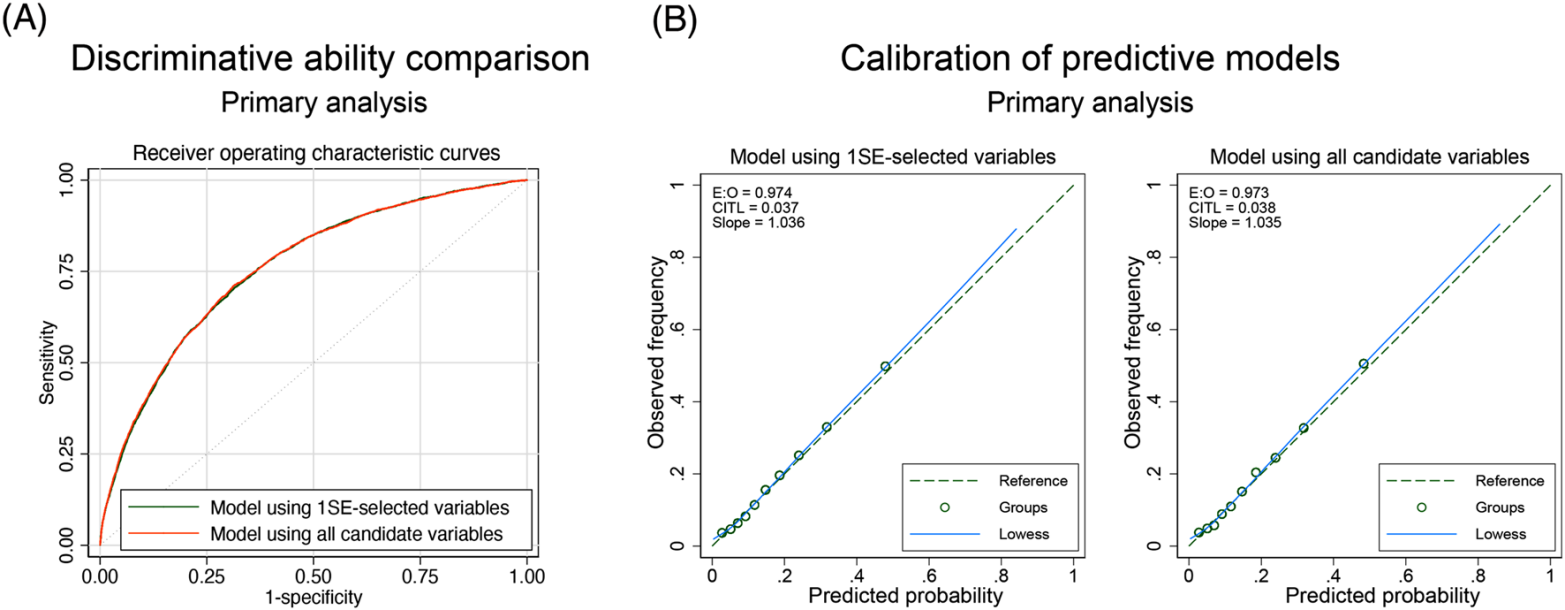
Receiver operating characteristic curve and calibration in primary analysis. (**A**) Receiver operating characteristic curves of the model using 1SE-selected variables and the model using all variables. (**B**) Calibration plots showing the models predicting non-home discharge among patients admitted for acute heart failure using the 1SE-selected variables and the model using all variables. *SE* standard-error, *E:O* estimated to observed ratio, *CITL* calibration in the large, *AUC* area under receiver operating curve, *CI* confidence interval

The model using 11 variables selected by 1SE rule of Lasso regression had a comparable discrimination ability with the model using all the 26 variables (*c*-statistic: 0.760 [95% CI 0.752–0.767] vs. 0.761 [95% CI 0.753–0.768], respectively) (Fig. 4A). The *c*-statistic of the model using each variable ordered by the variable importance, and *c*-statistic of the model as one single variable was added are shown in Supplementary Table 3. Analysis of calibration of both models showed that both models had a good calibration with an E:O ratio of 0.974, CITL value of 0.037, and slope of 1.036 in the model using the variables selected by 1SE of Lasso regression, and with an E:O ratio of 0.973, CITL value of 0.038, and slope of 1.035 in the model using all variables (Fig. 4B).

### Stratified analyses

When stratified by age and sex, the results were similar to those of the primary analysis. Among those aged ≥ 80 years (*n* = 69,168), 16,164 (23.4%) underwent transferal to another facility or in-hospital death, while among those aged < 80 years (*n* = 58,900), 6,166 (10.5%) underwent in-hospital death or non-home discharge. The age-stratified analyses selected the same predictors in the 1SE rule of Lasso regression completely although the order of the variable importance differed (Supplementary Figs. 2 and 3). The selected variables were Barthel index decrease, age increase, absence of comorbid hypertension, vasopressor use, impaired consciousness, worst NYHA class, BMI decrease, failure to initiate enteral alimentation, and kidney failure or kidney replacement therapy receipt within 2 days. Supplementary Figs. 4 and 5 show the discrimination ability and calibration for each group, and the results were similar to those of the main analysis. The odds ratios in the model using 1SE-selected variables are shown in Supplementary Tables 4 and 5.

Among female patients (*n* = 56,839), 11,450 (20.1%) underwent transferal to another facility or in-hospital death; while among male patients (*n* = 71,229), 10,880 (15.3%) underwent in-hospital death or non-home discharge. The sex-stratified analyses also yielded a similar pattern of variable importance in the Lasso regression model (Supplementary Figs. 6 and 7). Commonly selected variables were Barthel index decrease, absence of comorbid hypertension, vasopressor use, impaired consciousness, worst NYHA class, BMI decrease, failure to initiate enteral alimentation, kidney failure, and age increase. Supplementary Figs. 8 and 9 show the receiver operating characteristic curve and calibration curves for each group, respectively, and the results were similar to those of the main analysis. The odds ratios in the model using 1SE-selected variables are shown in Supplementary Tables 6 and 7.

Similarly, among patients with Barthel index ≥ 60 and Barthel index < 60, the results were similar. While patients with Barthel index ≥ 60 (*n* = 66,301), 6329 (9.5%) underwent transferal to another facility or in-hospital death, among patients with Barthel index < 60 (*n* = 61,767) 16,001 (25.9%) underwent in-hospital death or non-home discharge. Figures for variable importance, discriminative ability and calibration in the Lasso regression model are shown in Supplementary Figs. 10–13. Commonly selected variables were Barthel index decrease, absence of comorbid hypertension, vasopressor use, impaired consciousness, worst NYHA class, BMI decrease, failure to initiate enteral alimentation, and age increase. The odds ratios in the model using 1SE-selected variables are shown in Supplementary Tables 8 and 9.

### Sensitivity analyses

When changing the time window to 2 days to 3 days, a total of 126,923 patients with 21,871 (17.2%) reaching the outcome were analyzed (Supplementary Fig. 14). Supplementary Fig. 15 shows the importance of variables in predicting non-home discharge, and nine variables were selected; and c-statistics were comparable (model using 1SE-selected variables: 0.759 (95% CI 0.751–0.767) vs model using all variables: 0.761 (95% CI 0.754–0.769)). Analysis of calibration of both models revealed that both models had a comparable calibration ability with an E:O ratio of 0.985, CITL value of 0.021, and slope of 1.030 in the model using variables selected by 1SE rule of Lasso regression, and with an E:O ratio of 0.985, CITL value of 0.021, and slope of 1.032 in the model using all variables (Supplementary Fig. 16). The odds ratios in the model using 1SE-selected variables are shown in Supplementary Table 10.

After recategorizing patients with length of stay ≥ 30 days into those undergoing non-home discharge as the second sensitivity analysis, 39,332 (30.7%) reached the outcome. The importance of variables in predicting non-home discharge in this sensitivity analysis is shown in Supplementary Fig. 17. In the model, 17 variables were selected, the variables added to those selected in the primary analysis were female, anemia, failure to initiate rehabilitation within 2 days, readmission history, admission on weekends or holidays, and ischemic heart disease; c-statistics were comparable (model using 1SE-selected variables: 0.702 (95% CI, 0.695–0.709) vs model using all variables: 0.702 (95% CI, 0.696–0.709)). Both models had a comparable calibration (Supplementary Fig. 18B). The odds ratios in the model using 1SE-selected variables are shown in Supplementary Table 11.

Finally, after exclusion of those undergoing in-hospital death (*n* = 7879), 14,451 (12.0%) did not return home after hospitalization out of the total 120,189 patients. Supplementary Fig. 19 shows the importance of variables in predicting non-home discharge in this case scenario. In the model, six variables were selected, and c-statistics were comparable (model using 1SE-selected variables: 0.733 (95% CI, 0.724–0.743) vs model using all variables: 0.740 (95% CI, 0.730–0.749)). Both models had a comparable calibration (Supplementary Fig. 20B). The odds ratios in the model using 1SE-selected variables are shown in Supplementary Table 12.

## Discussion

The present study using a nationwide inpatient database including 128,068 patients hospitalized for AHF, we succeeded in developing a parsimonious and easy-to-grasp model via machine learning, and the model using 11 variables selected by the 1SE rule of Lasso regression presented a discriminative ability and calibration comparable to the model using all the 26 variables to predict non-home discharge. The essential determinants associated with non-home discharge, which were selected consistently throughout all the analyses, were low scores in Barthel index, advanced age, absence of comorbid hypertension, impaired consciousness, failure to initiate enteral alimentation within 2 days and low body weight.

There have been so far only few studies focusing on discharge disposition in patients hospitalized for AHF [20–24]. Our analysis showed that approximately 80% of study population discharged to home. This percentage was in agreement with a result of a multicenter registry including 4056 patients hospitalized for AHF in Japan, demonstrating the non-home discharge rate of 13.0% [24]. In the prospective study and our study, higher ages, lower BMI are commonly identified as predictive factors for non-home discharge [24]. Our study, although using the retrospective design, had novelty in showing the variable importance of each predictive marker via machine learning usage and yielding statistical significance on variables such as kidney failure probably due to the large sample size of our cohort. On the other hand, non-home discharge rate was much lower than that previously reported in U.S. For example, the recent analysis using the Get With The Guidelines-Heart Failure registry showed that the proportion of patients discharged to home was approximately 75% [22]. We suppose that this difference largely attributed to the difference in medical care system and length of hospital stay between countries. In this study, the median length of hospital stay was 12 days, whereas 4 days in U.S. [2, 22]. Longer hospital stay of AHF patients in Japan would contribute to the low non-home discharge rate.

Factors identified as predictors of non-home discharge are mostly pathophysiologically plausible. Barthel Index is an established indicator for activities of daily living, and our results including a multitude of sensitivity analyses demonstrated that lower Barthel Index was consistently the strongest predictor of non-home discharge. Given that poor activities of daily living were reported to be associated with adverse clinical outcomes of AHF patients [25], our results are reasonable. As expected, older age also strongly predicted non-home discharge. Older HF patients were increasing in developed countries including Japan [11]. For example, we previously reported that the proportion of hospitalized AHF patients aged ≥ 80 years and those aged ≥ 90 years in Japan reached 54.3% and 14.4%, respectively [11]. Given this background, older AHF patients at high risk for non-home discharge are expected to increase further. It is indispensable to establish regional health care system and network for older AHF patients who could not be discharged home. Low BMI was associated with an elevated risk for non-home discharge. Given that low Barthel Index, older age, and low BMI all increased the risk of non-home discharge, we may need to consider the potential role of malnutrition, frailty, or sarcopenia which would frequently coexist with these clinical characteristics in patients with AHF, for non-home discharge. It is also essential that the factors reflecting the severity at the hospital admission (e.g., advanced HF symptom [higher NYHA class], conscious disturbance, unstable hemodynamic state [requiring inotropic agent]) were identified as predictors for non-home discharge, which emphasized the clinical importance of early detection of worsening HF and timely treatment. Interestingly, we found that early enteral alimentation would decrease the possibility of non-home discharge, which was in line with our previous study [26]. Physicians may need to start the enteral alimentation for hospitalized patients with AHF as early as possible after the hospital admission in possible cases. At least, we should avoid delaying the initiation of feeding without a solid reason. Absence of comorbid hypertension was also a positive predictor of non-home discharge; this was plausible because results of previous studies [27, 28] showed that patients admitted for AHF with preserved or high blood pressure have generally a better prognosis than those with low blood pressure.

We applied a type of machine learning, Lasso regression, that stresses inference rather than prediction. Generally speaking, which machine learning method to choose in clinical epidemiology depends on the trade-off between interpretability versus complexity [15]. The trade-off means that the higher complexity a machine learning has, the lower interpretability but higher predictive ability it has while the lower complexity a machine learning has, the higher interpretability but lower predictive ability it has [15]. Lasso regression shrinks the coefficients of some noninformative variables to 0, and enhances the interpretability of the prepared model via this variable reduction; thus, Lasso regression is applied when researchers want to stress interpretability at the cost of predictive ability. Further, to strengthen variable shrinkage, we applied 1-SE rule of the lasso regression, which enabled identification of essential variables for the outcome of our study.

The present study has clinical implications. Our study showed that we could predict the discharge disposition of hospitalized patients owing to AHF with a high accuracy using the clinical information within 2 days after hospital admission which was generally included in an administrative claims database. For patients who are expected to have difficulty in being discharged home according to this prediction model, coordination of a medical facility where a patient would be transferred after the acute care for AHF should be started early in the hospitalization process to reduce the burden on healthcare professionals, patients, and their families, and to enable smooth healthcare coordination in a community. Furthermore, we created a simple model, whereby clinicians can easily apply to real-world clinical practice.

We acknowledge several limitations to the present study. The possibility of unmeasured residual confounding could not be eliminated. For example, the presence of family caregivers would influence the discharge disposition. Further, we could not utilize and adjust for not only biomarkers such as blood pressure, BNP, electrolytes, hemoglobin, or serum creatinine but also data on etiology of HF or echocardiographic parameters such as left ventricular ejection fraction. Unfortunately, these data were unavailable in our DPC database. Although the validity of diagnoses and procedures in this database was reported to be high in Japan [29, 30], the recorded diagnoses of might have been misclassified. Healthcare system and regional factors could affect the study results. For example, Japan has the universal healthcare insurance system and patients could always get medical service with relatively low cost. Therefore, although this study used a nationwide large-scale inpatient dataset, our results cannot be generalized to other countries. Especially, validation of our predictive model needs conducting with use of different databases in different countries or settings. Further investigations using other independent datasets are required to validate our result.

## Conclusions

Our analysis of a nationwide inpatient database demonstrated that approximately 80% of hospitalized AHF patients discharged to home, whereas the remaining 20% of patients underwent in-hospital death or discharged to other facilities including other medical institutes and nursing homes. We developed a machine learning-based prediction model, and low scores in activities of daily living, advanced age, absence of comorbid hypertension, impaired consciousness, failure to initiate enteral alimentation within 2 days and low body weight were strong determinants of non-home discharge among hospitalized patients for AHF. We believe that results of the present study could provide practical data to build up the optimal medical care system and network for HF patients in the era when patients with HF are rapidly increasing worldwide.

### Supplementary Information

Below is the link to the electronic supplementary material.

Supplementary file1 (DOCX 40 KB)

Supplementary file1 (PPTX 14191 KB)

## Data Availability

The datasets analyzed during the current study are not publicly available due to contracts with the hospitals providing data to the database.
