# Supplementary material for: A machine-learning-based prediction of non-home discharge among acute heart failure patients
Source: Clin Res Cardiol. 2023 May 3;113(4):522–32. doi: 10.1007/s00392-023-02209-0 (PMC10955024; doi:10.1007/s00392-023-02209-0)
Supplement: Supplementary file 1 — Supplementary file1 (DOCX 40 KB) [file 392_2023_2209_MOESM1_ESM.docx]

**Supplementary Table 1.** Characteristics of eligible patients divided by whether to be a training or validation set

| Variable | Category | **Training** | **Validation** | **p-value** |
| --- | --- | --- | --- | --- |
|  |  | **n=102,454** | **n=25,614** |  |
| Age |  | 81.0 (72.0-87.0) | 80.0 (72.0-87.0) | 0.46 |
| Age category | <65 years | 12,941 (12.6%) | 3,193 (12.5%) | 0.048 |
|  | 65-74 years | 18,710 (18.3%) | 4,861 (19.0%) |  |
|  | 75-84 years | 36,339 (35.5%) | 8,944 (34.9%) |  |
|  | 85- years | 34,464 (33.6%) | 8,616 (33.6%) |  |
| Male |  | 57,099 (55.7%) | 14,130 (55.2%) | 0.10 |
| Body mass index (kg/m^2^) |  | 22.2 (19.7-25.2) | 22.2 (19.7-25.1) | 0.37 |
| Body mass index category | -18.49 kg/m^2^ | 15,733 (15.4%) | 4,007 (15.6%) | 0.51 |
|  | 18.5-24.99 kg/m^2^ | 60,121 (58.7%) | 14,995 (58.5%) |  |
|  | 25.00- kg/m^2^ | 26,600 (26.0%) | 6,612 (25.8%) |  |
| Barthel index |  | 60 (15-100) | 60 (10-100) | 0.69 |
| Consciousness on admission | Alert | 88,556 (86.4%) | 22,135 (86.4%) | 0.48 |
|  | Impaired | Impaired | 13,898 (13.6%) |  |
| Categorization based on New York Heart Association | Class II | 28,287 (27.6%) | 7,216 (28.2%) | 0.20 |
|  | Class III | 39,607 (38.7%) | 9,814 (38.3%) |  |
|  | Class IV | 34,560 (33.7%) | 8,584 (33.5%) |  |
| Admission on weekends or holidays |  | 22,625 (22.1%) | 5,676 (22.2%) | 0.79 |
| With readmission history for heart failure within previous 30 days |  | 7,398 (7.2%) | 1,917 (7.5%) | 0.15 |
| Hypertension |  | 56,642 (55.3%) | 14,312 (55.9%) | 0.089 |
| Diabetes mellitus |  | 29,327 (28.6%) | 7,312 (28.5%) | 0.81 |
| Kidney failure or kidney replacement therapy receipt within 2 days |  | 13,703 (13.4%) | 3,437 (13.4%) | 0.85 |
| Liver disease |  | 2,324 (2.3%) | 610 (2.4%) | 0.28 |
| Pulmonary disease |  | 7,762 (7.6%) | 1,895 (7.4%) | 0.34 |
| Ischemic heart disease |  | 1,418 (1.4%) | 308 (1.2%) | 0.024 |
| Cancer |  | 4,688 (4.6%) | 1,207 (4.7%) | 0.35 |
| Dilated cardiomyopathy |  | 4,822 (4.7%) | 1,243 (4.9%) | 0.32 |
| Shock or ventricular fibrillation on admission |  | 1,295 (1.3%) | 291 (1.1%) | 0.098 |
| Osteoporosis |  | 1,868 (1.8%) | 497 (1.9%) | 0.21 |
| Charlson comorbidity index |  | 0.0 (0.0-1.0) | 0.0 (0.0-1.0) | 0.91 |
| Anemia on admission or transfusion within 2 days |  | 8,597 (8.4%) | 2,238 (8.7%) | 0.075 |
| Intensive cardiopulmonary support within 2 days |  | 15,146 (14.8%) | 3,818 (14.9%) | 0.62 |
| Intravenous loop diuretics within 2 days |  | 71,398 (69.7%) | 17,870 (69.8%) | 0.81 |
| Intravenous vasopressors within 2 days |  | 16,010 (15.6%) | 3,941 (15.4%) | 0.34 |
| Intensive care unit use within 2 days |  | 17,614 (17.2%) | 4,446 (17.4%) | 0.53 |
| Enteral alimentation within 2 days |  | 90,104 (87.9%) | 22,580 (88.2%) | 0.36 |
| Rehabilitation within 2 days |  | 11,808 (11.5%) | 2,976 (11.6%) | 0.68 |
| Non-home discharge or in-hospital death |  | 17,784 (17.4%) | 4,546 (17.7%) | 0.14 |

Data are presented as median (interquartile range) for continuous measures, and n (%) for categorical measures.

**Supplementary Table 2.** Odds ratios of in the model using all the variables in predicting non-home discharge or in-hospital death

| Variable | Category | Odds ratio | 95% confidence interval | | | P value |
| --- | --- | --- | --- | --- | --- | --- |
| Age (10-year increase) |  | 1.47 | 1.44 | - | 1.50 | <0.001 |
| Sex | Female | Reference |  |  |  |  |
|  | Male | 0.97 | 0.94 | - | 1.01 | 0.14 |
| Body mass index (5-kg/m^2^ decrease) | | 1.19 | 1.16 | - | 1.22 | <0.001 |
| Barthel index (10-point decrease) | | 1.12 | 1.12 | - | 1.13 | <0.001 |
| Consciousness on admission | Alert | Reference |  |  |  |  |
|  | Impaired | 1.59 | 1.52 | - | 1.67 | <0.001 |
| Categorization based on New York Heart Association | Class II | Reference |  |  |  |  |
|  | Class III | 1.27 | 1.21 | - | 1.33 | <0.001 |
|  | Class IV | 1.68 | 1.60 | - | 1.77 | <0.001 |
| Admission on weekends or holidays |  | 0.94 | 0.91 | - | 0.98 | 0.007 |
| With readmission history for heart failure within previous 30 days |  | 1.11 | 1.04 | - | 1.19 | 0.001 |
| Absence of comorbid hypertension | | 1.78 | 1.72 | - | 1.84 | <0.001 |
| Diabetes mellitus |  | 0.92 | 0.88 | - | 0.95 | <0.001 |
| Kidney failure or renal kidney replacement therapy receipt within 2 days |  | 1.23 | 1.16 | - | 1.29 | <0.001 |
| Liver disease |  | 0.94 | 0.83 | - | 1.06 | 0.30 |
| Pulmonary disease |  | 0.85 | 0.79 | - | 0.91 | <0.001 |
| Ischemic heart disease |  | 1.39 | 1.22 | - | 1.59 | <0.001 |
| Cancer |  | 0.91 | 0.83 | - | 1.00 | 0.048 |
| Dilated cardiomyopathy |  | 0.86 | 0.78 | - | 0.95 | 0.002 |
| Shock or ventricular fibrillation on admission |  | 1.47 | 1.29 | - | 1.68 | <0.001 |
| Osteoporosis |  | 0.68 | 0.59 | - | 0.78 | <0.001 |
| Charlson comorbidity index |  | 1.09 | 1.07 | - | 1.12 | <0.001 |
| Anemia on admission or transfusion within 2 days |  | 1.05 | 0.99 | - | 1.11 | 0.093 |
| Intensive cardiopulmonary support within 2 days |  | 0.86 | 0.82 | - | 0.90 | <0.001 |
| Intravenous loop diuretics within 2 days |  | 0.89 | 0.86 | - | 0.93 | <0.001 |
| Intravenous vasopressors within 2 days |  | 1.61 | 1.54 | - | 1.68 | <0.001 |
| Intensive care unit use within 2 days |  | 0.96 | 0.92 | - | 1.01 | 0.093 |
| Failure to initiate enteral alimentation within 2 days | | 1.57 | 1.50 | - | 1.65 | <0.001 |
| Failure to initiate rehabilitation within 2 days | | 1.06 | 1.00 | - | 1.12 | 0.042 |

**Supplementary Table 3.** C-statistic of models using each variable and each variable addition, ordered by the variable importance

| Variable used in models | C-statistic in the model  using single variable | | | | C-statistic in the model  after the variable addition | | | |
| --- | --- | --- | --- | --- | --- | --- | --- | --- |
|  | C-statistic | 95% confidence interval | | | C-statistic | 95% confidence interval | | |
| Barthel index | 0.683 | 0.675 | - | 0.691 |  |  |  |  |
| Age | 0.656 | 0.647 | - | 0.665 | 0.717 | 0.709 | - | 0.726 |
| Absence of comorbid hypertension | 0.586 | 0.578 | - | 0.594 | 0.733 | 0.725 | - | 0.741 |
| Consciousness on admission | 0.580 | 0.573 | - | 0.586 | 0.739 | 0.731 | - | 0.747 |
| Intravenous vasopressors within 2 days | 0.545 | 0.539 | - | 0.552 | 0.746 | 0.738 | - | 0.754 |
| Categorization based on New York Heart Association | 0.593 | 0.585 | - | 0.601 | 0.752 | 0.745 | - | 0.760 |
| Body mass index | 0.600 | 0.591 | - | 0.609 | 0.754 | 0.747 | - | 0.762 |
| Failure to initiate enteral alimentation within 2 days | 0.559 | 0.552 | - | 0.565 | 0.757 | 0.750 | - | 0.765 |
| Kidney failure or kidney replacement therapy receipt within 2 days | 0.527 | 0.521 | - | 0.533 | 0.759 | 0.751 | - | 0.766 |
| Charlson comorbidity index | 0.532 | 0.524 | - | 0.540 | 0.759 | 0.752 | - | 0.767 |
| Shock or ventricular fibrillation on admission | 0.507 | 0.504 | - | 0.509 | 0.760 | 0.752 | - | 0.767 |

**Supplementary Table 4.** Odds ratios of the variables selected by 1 standard-error rule of Lasso regression in predicting non-home discharge or in-hospital death among patients aged ≥ 80 years

| Variable | Category | Odds ratio | 95% confidence interval | | | P value |
| --- | --- | --- | --- | --- | --- | --- |
| Age (10-year increase) | | 1.62 | 1.55 | - | 1.70 | <0.001 |
| Body mass index (5-kg/m^2^ decrease) | | 1.20 | 1.17 | - | 1.23 | <0.001 |
| Barthel index (10-point decrease) | | 1.12 | 1.12 | - | 1.13 | <0.001 |
| Consciousness on admission | Alert | Reference |  |  |  |  |
|  | Impaired | 1.59 | 1.51 | - | 1.67 | <0.001 |
| Categorization based on New York Heart Association | Class II | Reference |  |  |  |  |
|  | Class III | 1.21 | 1.15 | - | 1.28 | <0.001 |
|  | Class IV | 1.57 | 1.48 | - | 1.66 | <0.001 |
| Absence of comorbid hypertension | | 1.71 | 1.64 | - | 1.78 | <0.001 |
| Kidney failure or kidney replacement therapy receipt within 2 days | | 1.40 | 1.32 | - | 1.48 | <0.001 |
| Intravenous vasopressors within 2 days | | 1.63 | 1.54 | - | 1.73 | <0.001 |
| Failure to initiate enteral alimentation within 2 days |  | 1.60 | 1.51 | - | 1.70 | <0.001 |

**Supplementary Table 5.** Odds ratios of the variables selected by 1 standard-error rule of Lasso regression in predicting non-home discharge or in-hospital death among patients aged < 80 years

| Variable | Category | Odds ratio | 95% confidence interval | | | P value |
| --- | --- | --- | --- | --- | --- | --- |
| Age (10-year increase) | | 1.18 | 1.14 | - | 1.22 | <0.001 |
| Body mass index (5-kg/m^2^ decrease) | | 1.21 | 1.16 | - | 1.25 | <0.001 |
| Barthel index (10-point decrease) | | 1.10 | 1.09 | - | 1.11 | <0.001 |
| Consciousness on admission | Alert | Reference |  |  |  |  |
|  | Impaired | 1.61 | 1.47 | - | 1.75 | <0.001 |
| Categorization based on New York Heart Association | Class II | Reference |  |  |  |  |
|  | Class III | 1.46 | 1.34 | - | 1.60 | <0.001 |
|  | Class IV | 1.87 | 1.71 | - | 2.04 | <0.001 |
| Absence of comorbid hypertension | | 2.11 | 1.98 | - | 2.25 | <0.001 |
| Kidney failure or kidney replacement therapy receipt within 2 days | | 1.42 | 1.31 | - | 1.54 | <0.001 |
| Shock or ventricular fibrillation on admission | | 1.62 | 1.35 | - | 1.95 | <0.001 |
| Intravenous vasopressors within 2 days | | 1.64 | 1.53 | - | 1.76 | <0.001 |
| Failure to initiate enteral alimentation within 2 days |  | 1.45 | 1.34 | - | 1.57 | <0.001 |

**Supplementary Table 6.** Odds ratios of the variables selected by 1 standard-error rule of Lasso regression in predicting non-home discharge or in-hospital death among female patients

| Variable | Category | Odds ratio | 95% confidence interval | | | P value |
| --- | --- | --- | --- | --- | --- | --- |
| Age (10-year increase) | | 1.55 | 1.51 | - | 1.60 | <0.001 |
| Body mass index (5-kg/m^2^ decrease) | | 1.15 | 1.12 | - | 1.19 | <0.001 |
| Barthel index (10-point decrease) | | 1.12 | 1.11 | - | 1.13 | <0.001 |
| Consciousness on admission | Alert | Reference |  |  |  |  |
|  | Impaired | 1.48 | 1.40 | - | 1.58 | <0.001 |
| Categorization based on New York Heart Association | Class II | Reference |  |  |  |  |
|  | Class III | 1.22 | 1.14 | - | 1.30 | <0.001 |
|  | Class IV | 1.53 | 1.43 | - | 1.63 | <0.001 |
| Absence of comorbid hypertension | | 1.77 | 1.69 | - | 1.86 | <0.001 |
| Kidney failure or kidney replacement therapy receipt within 2 days | | 1.29 | 1.20 | - | 1.39 | <0.001 |
| Charlson comorbidity index | | 1.09 | 1.06 | - | 1.11 | <0.001 |
| Intravenous vasopressors within 2 days | | 1.54 | 1.44 | - | 1.65 | <0.001 |
| Failure to initiate enteral alimentation within 2 days |  | 1.63 | 1.52 | - | 1.74 | <0.001 |

**Supplementary Table 7.** Odds ratios of the variables selected by 1 standard-error rule of Lasso regression in predicting non-home discharge or in-hospital death among male patients

| Variable | Category | Odds ratio | 95% confidence interval | | | P value |
| --- | --- | --- | --- | --- | --- | --- |
| Age (10-year increase) | | 1.42 | 1.38 | - | 1.46 | <0.001 |
| Body mass index (5-kg/m^2^ decrease) | | 1.27 | 1.23 | - | 1.32 | <0.001 |
| Barthel index (10-point decrease) | | 1.11 | 1.10 | - | 1.12 | <0.001 |
| Consciousness on admission | Alert | Reference |  |  |  |  |
|  | Impaired | 1.69 | 1.58 | - | 1.80 | <0.001 |
| Categorization based on New York Heart Association | Class II | Reference |  |  |  |  |
|  | Class III | 1.34 | 1.25 | - | 1.43 | <0.001 |
|  | Class IV | 1.77 | 1.66 | - | 1.90 | <0.001 |
| Absence of comorbid hypertension | | 1.92 | 1.82 | - | 2.01 | <0.001 |
| Kidney failure or kidney replacement therapy receipt within 2 days | | 1.38 | 1.30 | - | 1.47 | <0.001 |
| Shock or ventricular fibrillation on admission | | 1.66 | 1.40 | - | 1.98 | <0.001 |
| Intravenous vasopressors within 2 days | | 1.67 | 1.57 | - | 1.77 | <0.001 |
| Failure to initiate enteral alimentation within 2 days |  | 1.51 | 1.41 | - | 1.61 | <0.001 |

**Supplementary Table 8.** Odds ratios of the variables selected by 1 standard-error rule of Lasso regression in predicting non-home discharge or in-hospital death among patients with Barthel index ≥ 60

| Variable | Category | Odds ratio | 95% confidence interval | | | P value |
| --- | --- | --- | --- | --- | --- | --- |
| Age (10-year increase) | | 1.36 | 1.32 | - | 1.41 | <0.001 |
| Body mass index (5-kg/m^2^ decrease) | | 1.24 | 1.19 | - | 1.29 | <0.001 |
| Barthel index (10-point decrease) | | 1.16 | 1.14 | - | 1.18 | <0.001 |
| Consciousness on admission | Alert | Reference |  |  |  |  |
|  | Impaired | 1.76 | 1.58 | - | 1.95 | <0.001 |
| Categorization based on New York Heart Association | Class II | Reference |  |  |  |  |
|  | Class III | 1.37 | 1.27 | - | 1.48 | <0.001 |
|  | Class IV | 2.11 | 1.95 | - | 2.28 | <0.001 |
| Absence of comorbid hypertension | | 1.85 | 1.74 | - | 1.96 | <0.001 |
| Intravenous vasopressors within 2 days | | 1.76 | 1.63 | - | 1.90 | <0.001 |
| Failure to initiate enteral alimentation within 2 days |  | 1.86 | 1.69 | - | 2.05 | <0.001 |

**Supplementary Table 9.** Odds ratios of the variables selected by 1 standard-error rule of Lasso regression in predicting non-home discharge or in-hospital death among patients with Barthel index < 60

| Variable | Category | Odds ratio | 95% confidence interval | | | P value |
| --- | --- | --- | --- | --- | --- | --- |
| Age (10-year increase) | | 1.57 | 1.53 | - | 1.61 | <0.001 |
| Body mass index (5-kg/m^2^ decrease) | | 1.19 | 1.16 | - | 1.22 | <0.001 |
| Barthel index (10-point decrease) | | 1.14 | 1.12 | - | 1.15 | <0.001 |
| Consciousness on admission | Alert | Reference |  |  |  |  |
|  | Impaired | 1.56 | 1.49 | - | 1.64 | <0.001 |
| Categorization based on New York Heart Association | Class II | Reference |  |  |  |  |
|  | Class III | 1.21 | 1.14 | - | 1.28 | <0.001 |
|  | Class IV | 1.43 | 1.35 | - | 1.52 | <0.001 |
| Absence of comorbid hypertension | | 1.27 | 1.20 | - | 1.35 | <0.001 |
| Kidney failure or kidney replacement therapy receipt within 2 days | | 1.79 | 1.71 | - | 1.87 | <0.001 |
| Charlson comorbidity index | | 1.07 | 1.05 | - | 1.09 | <0.001 |
| Shock or ventricular fibrillation on admission | | 1.55 | 1.33 | - | 1.81 | <0.001 |
| Intravenous vasopressors within 2 days | | 1.53 | 1.45 | - | 1.61 | <0.001 |
| Failure to initiate enteral alimentation within 2 days | | 1.46 | 1.38 | - | 1.54 | <0.001 |

**Supplementary Table 10.** Odds ratios of the variables selected by 1 standard-error rule of Lasso regression in predicting non-home discharge or in-hospital death when time window was changed from 2 days to 3 days (sensitivity analysis 1)

| Variable | Category | Odds ratio | 95% confidence interval | | | P value |
| --- | --- | --- | --- | --- | --- | --- |
| Age (10-year increase) | | 1.50 | 1.47 | - | 1.53 | <0.001 |
| Body mass index (5-kg/m^2^ decrease) | | 1.20 | 1.17 | - | 1.23 | <0.001 |
| Barthel index (10-point decrease) | | 1.12 | 1.11 | - | 1.12 | <0.001 |
| Consciousness on admission | Alert | Reference |  |  |  |  |
|  | Impaired | 1.60 | 1.53 | - | 1.67 | <0.001 |
| Categorization based on New York Heart Association | Class II | Reference |  |  |  |  |
|  | Class III | 1.25 | 1.19 | - | 1.31 | <0.001 |
|  | Class IV | 1.59 | 1.52 | - | 1.67 | <0.001 |
| Absence of comorbid hypertension | | 1.82 | 1.75 | - | 1.88 | <0.001 |
| Kidney failure or kidney replacement therapy receipt within 3 days | | 1.37 | 1.31 | - | 1.44 | <0.001 |
| Intravenous vasopressors within 3 days | | 1.70 | 1.62 | - | 1.77 | <0.001 |
| Failure to initiate enteral alimentation within 3 days |  | 1.91 | 1.80 | - | 2.02 | <0.001 |

**Supplementary Table 11.** Odds ratios of the variables selected by 1 standard-error rule of Lasso regression in predicting non-home discharge or in-hospital death when regarding patients with length of stay ≥ 30 days as those undergoing non-home discharge (sensitivity analysis 2)

| Variable | Category | Odds ratio | 95% confidence interval | | | P value |
| --- | --- | --- | --- | --- | --- | --- |
| Age (10-year increase) | | 1.21 | 1.20 | - | 1.23 | <0.001 |
| Sex | Male | 0.89 | 0.86 | - | 0.91 | <0.001 |
| Body mass index (5-kg/m^2^ decrease) | | 1.09 | 1.08 | - | 1.11 | <0.001 |
| Barthel index (10-point decrease) | | 1.09 | 1.08 | - | 1.11 | <0.001 |
| Consciousness on admission | Alert | Reference |  |  |  |  |
|  | Impaired | 1.37 | 1.32 | - | 1.43 | <0.001 |
| Categorization based on New York Heart Association | Class II | Reference |  |  |  |  |
|  | Class III | 1.24 | 1.20 | - | 1.29 | <0.001 |
|  | Class IV | 1.46 | 1.41 | - | 1.52 | <0.001 |
| Admission on weekends or holidays | | 0.85 | 0.82 | - | 0.88 | <0.001 |
| With readmission history for heart failure within previous 30 days | | 1.20 | 1.14 | - | 1.26 | <0.001 |
| Absence of comorbid hypertension | | 1.63 | 1.58 | - | 1.67 | <0.001 |
| Kidney failure or kidney replacement therapy receipt within 2 days | | 1.30 | 1.25 | - | 1.35 | <0.001 |
| Charlson comorbidity index | | 1.08 | 1.07 | - | 1.09 | <0.001 |
| Ischemic heart disease | | 1.44 | 1.28 | - | 1.61 | <0.001 |
| Shock or ventricular fibrillation on admission | | 1.37 | 1.22 | - | 1.55 | <0.001 |
| Anemia on admission or transfusion within 2 days | | 1.17 | 1.11 | - | 1.22 | <0.001 |
| Intravenous vasopressors within 2 days | | 1.78 | 1.72 | - | 1.85 | <0.001 |
| Failure to initiate enteral alimentation within 2 days | | 1.71 | 1.64 | - | 1.78 | <0.001 |
| Failure to initiate rehabilitation within 2 days | | 1.21 | 1.16 | - | 1.27 | <0.001 |

**Supplementary Table 12.** Odds ratios of the variables selected by 1 standard-error rule of Lasso regression in predicting non-home discharge or in-hospital death when excluding those undergoing in-hospital death (sensitivity analysis 3)

| Variable | Category | Odds ratio | 95% confidence interval | | | P value |
| --- | --- | --- | --- | --- | --- | --- |
| Age (10-year increase) | | 1.42 | 1.39 | - | 1.46 | <0.001 |
| Body mass index (5-kg/m^2^ decrease) | | 1.17 | 1.14 | - | 1.20 | <0.001 |
| Barthel index (10-point decrease) | | 1.13 | 1.13 | - | 1.14 | <0.001 |
| Consciousness on admission | Alert | Reference |  |  |  |  |
|  | Impaired | 1.66 | 1.58 | - | 1.75 | <0.001 |
| Absence of comorbid hypertension | | 1.62 | 1.56 | - | 1.69 | <0.001 |
| Failure to initiate enteral alimentation within 2 days | | 1.42 | 1.35 | - | 1.51 | <0.001 |
