# Supplementary material for: A machine-learning-based prediction of non-home discharge among acute heart failure patients
Source: Clin Res Cardiol. 2023 May 3;113(4):522–32. doi: 10.1007/s00392-023-02209-0 (PMC10955024; doi:10.1007/s00392-023-02209-0)
Supplement: Supplementary file 2 — Supplementary file1 (PPTX 14191 KB) [file 392_2023_2209_MOESM2_ESM.pptx]

## Slide 1
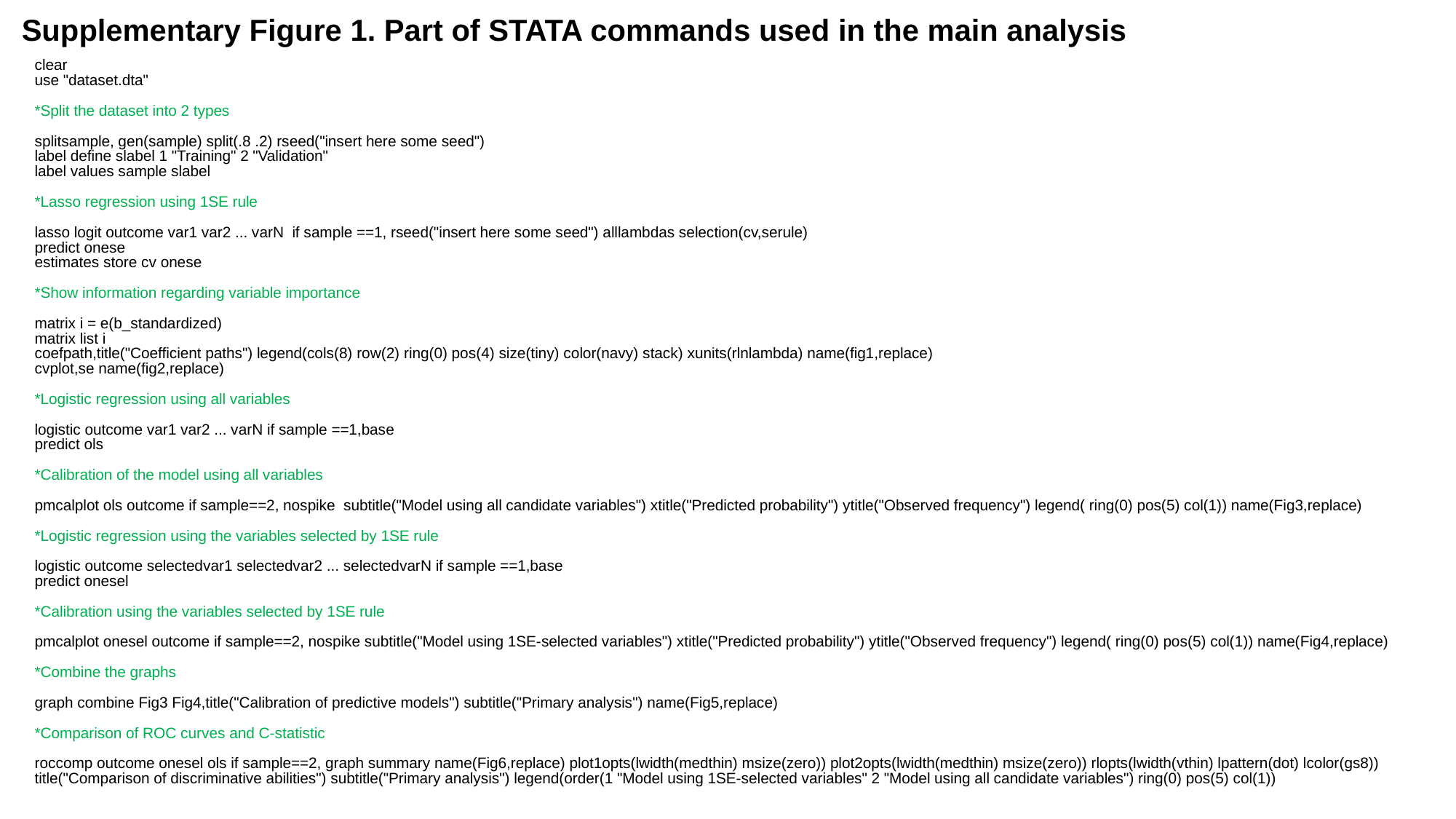

Supplementary Figure 1. Part of STATA commands used in the main analysis
clear
use "dataset.dta"
*Split the dataset into 2 types
splitsample, gen(sample) split(.8 .2) rseed("insert here some seed")
label define slabel 1 "Training" 2 "Validation"
label values sample slabel
*Lasso regression using 1SE rule
lasso logit outcome var1 var2 ... varN if sample ==1, rseed("insert here some seed") alllambdas selection(cv,serule)
predict onese
estimates store cv onese
*Show information regarding variable importance
matrix i = e(b_standardized)
matrix list i
coefpath,title("Coefficient paths") legend(cols(8) row(2) ring(0) pos(4) size(tiny) color(navy) stack) xunits(rlnlambda) name(fig1,replace)
cvplot,se name(fig2,replace)
*Logistic regression using all variables
logistic outcome var1 var2 ... varN if sample ==1,base
predict ols
*Calibration of the model using all variables
pmcalplot ols outcome if sample==2, nospike subtitle("Model using all candidate variables") xtitle("Predicted probability") ytitle("Observed frequency") legend( ring(0) pos(5) col(1)) name(Fig3,replace)
*Logistic regression using the variables selected by 1SE rule
logistic outcome selectedvar1 selectedvar2 ... selectedvarN if sample ==1,base
predict onesel
*Calibration using the variables selected by 1SE rule
pmcalplot onesel outcome if sample==2, nospike subtitle("Model using 1SE-selected variables") xtitle("Predicted probability") ytitle("Observed frequency") legend( ring(0) pos(5) col(1)) name(Fig4,replace)
*Combine the graphs
graph combine Fig3 Fig4,title("Calibration of predictive models") subtitle("Primary analysis") name(Fig5,replace)
*Comparison of ROC curves and C-statistic
roccomp outcome onesel ols if sample==2, graph summary name(Fig6,replace) plot1opts(lwidth(medthin) msize(zero)) plot2opts(lwidth(medthin) msize(zero)) rlopts(lwidth(vthin) lpattern(dot) lcolor(gs8)) title("Comparison of discriminative abilities") subtitle("Primary analysis") legend(order(1 "Model using 1SE-selected variables" 2 "Model using all candidate variables") ring(0) pos(5) col(1))

## Slide 2
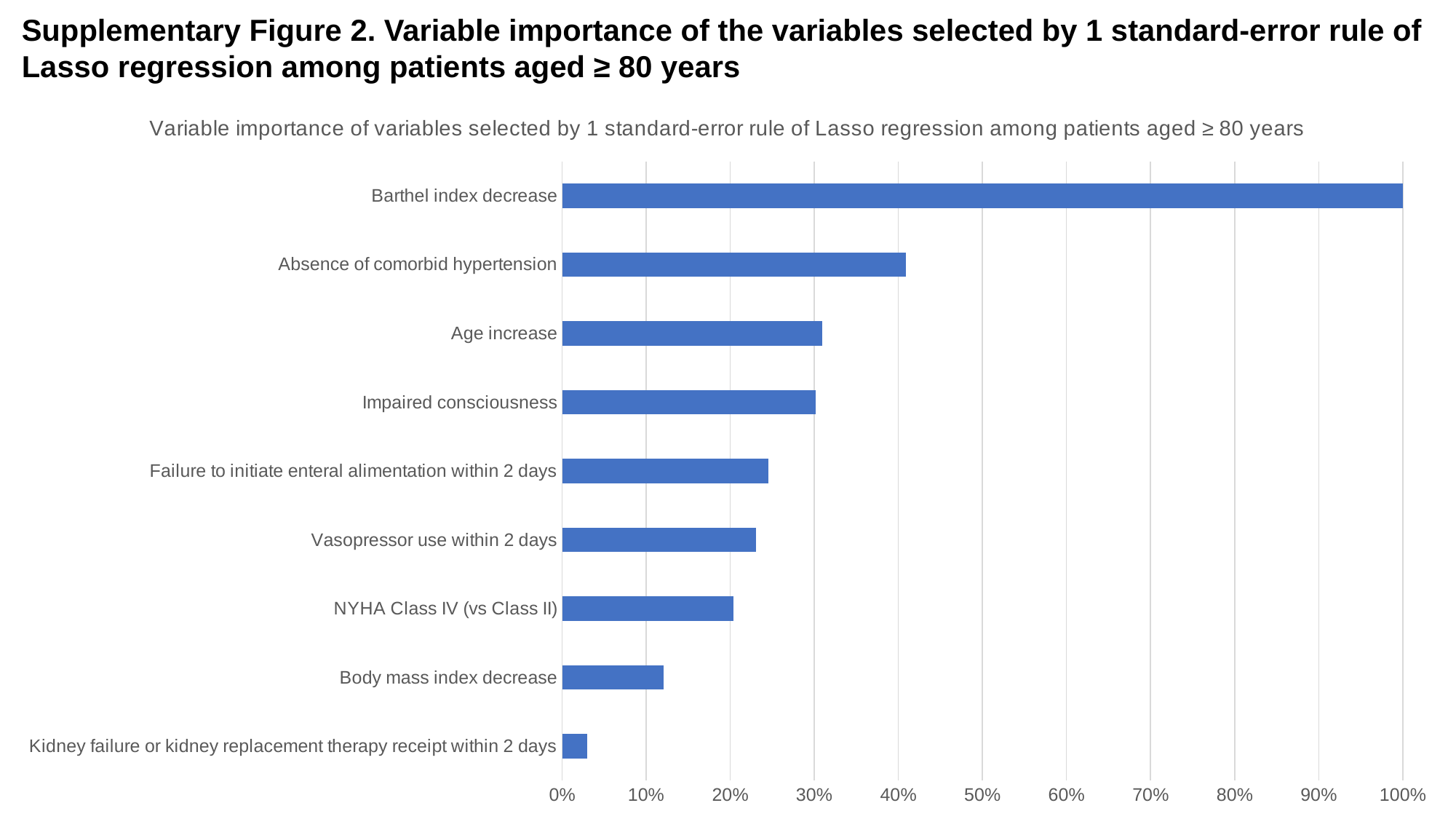

Supplementary Figure 2. Variable importance of the variables selected by 1 standard-error rule of Lasso regression among patients aged ≥ 80 years
### Chart: Variable importance of variables selected by 1 standard-error rule of Lasso regression among patients aged ≥ 80 years
| Category | |
|---|---|
| Kidney failure or kidney replacement therapy receipt within 2 days | 0.030255985966215166 |
| Body mass index decrease | 0.12079463204879146 |
| NYHA Class IV (vs Class II) | 0.2039145124566578 |
| Vasopressor use within 2 days | 0.23088091842307104 |
| Failure to initiate enteral alimentation within 2 days | 0.24555360579067143 |
| Impaired consciousness | 0.30135077440173275 |
| Age increase | 0.30940930283874507 |
| Absence of comorbid hypertension | 0.4088487620246458 |
| Barthel index decrease | 1.0 |

## Slide 3
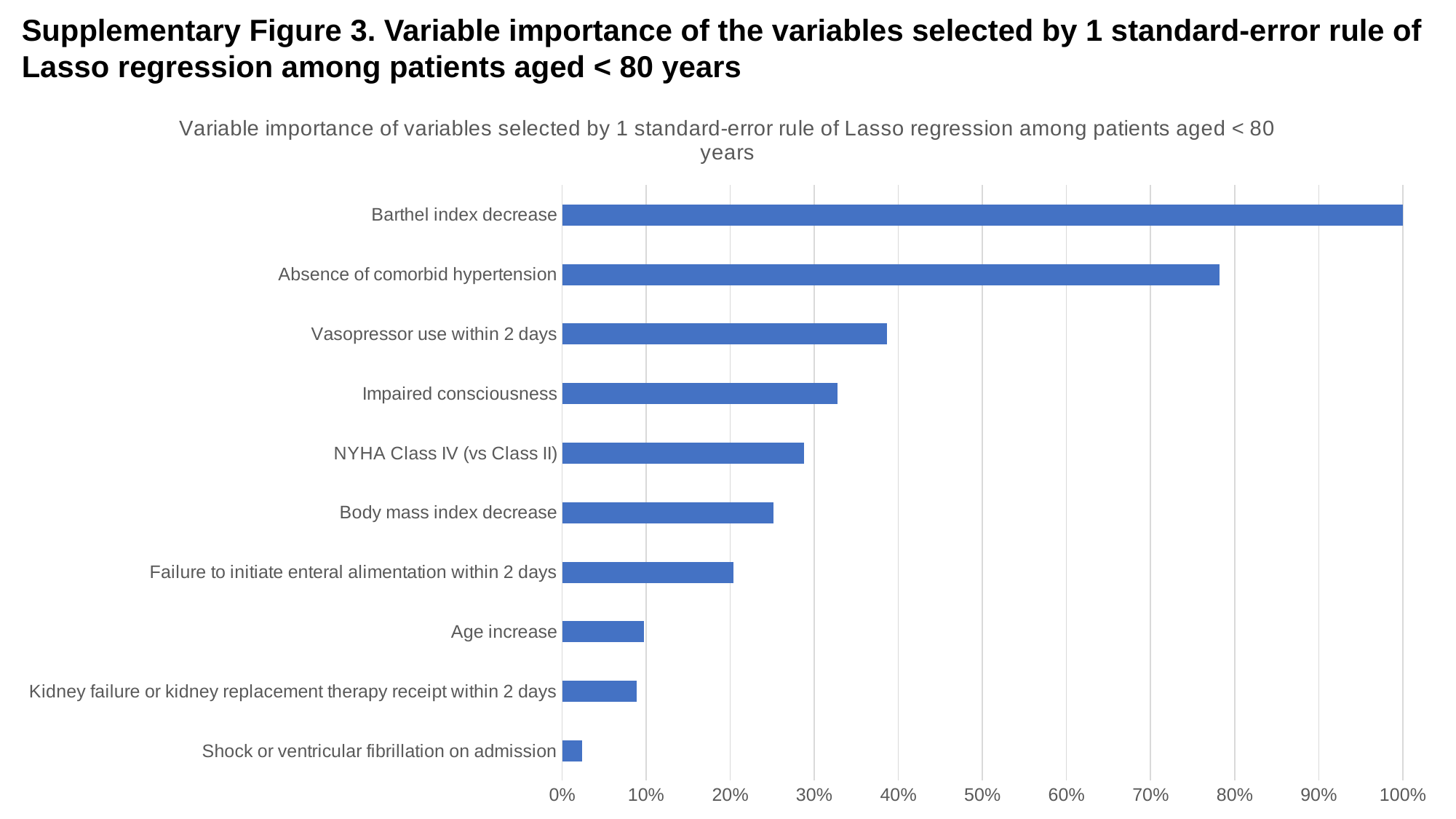

Supplementary Figure 3. Variable importance of the variables selected by 1 standard-error rule of Lasso regression among patients aged < 80 years
### Chart: Variable importance of variables selected by 1 standard-error rule of Lasso regression among patients aged < 80 years
| Category | |
|---|---|
| Shock or ventricular fibrillation on admission | 0.024005519275748212 |
| Kidney failure or kidney replacement therapy receipt within 2 days | 0.08903850976467954 |
| Age increase | 0.09778356640038785 |
| Failure to initiate enteral alimentation within 2 days | 0.20402107637215303 |
| Body mass index decrease | 0.2517437918907262 |
| NYHA Class IV (vs Class II) | 0.28825966359285343 |
| Impaired consciousness | 0.32802277567936744 |
| Vasopressor use within 2 days | 0.3863552386119451 |
| Absence of comorbid hypertension | 0.7820038937559601 |
| Barthel index decrease | 1.0 |

## Slide 4
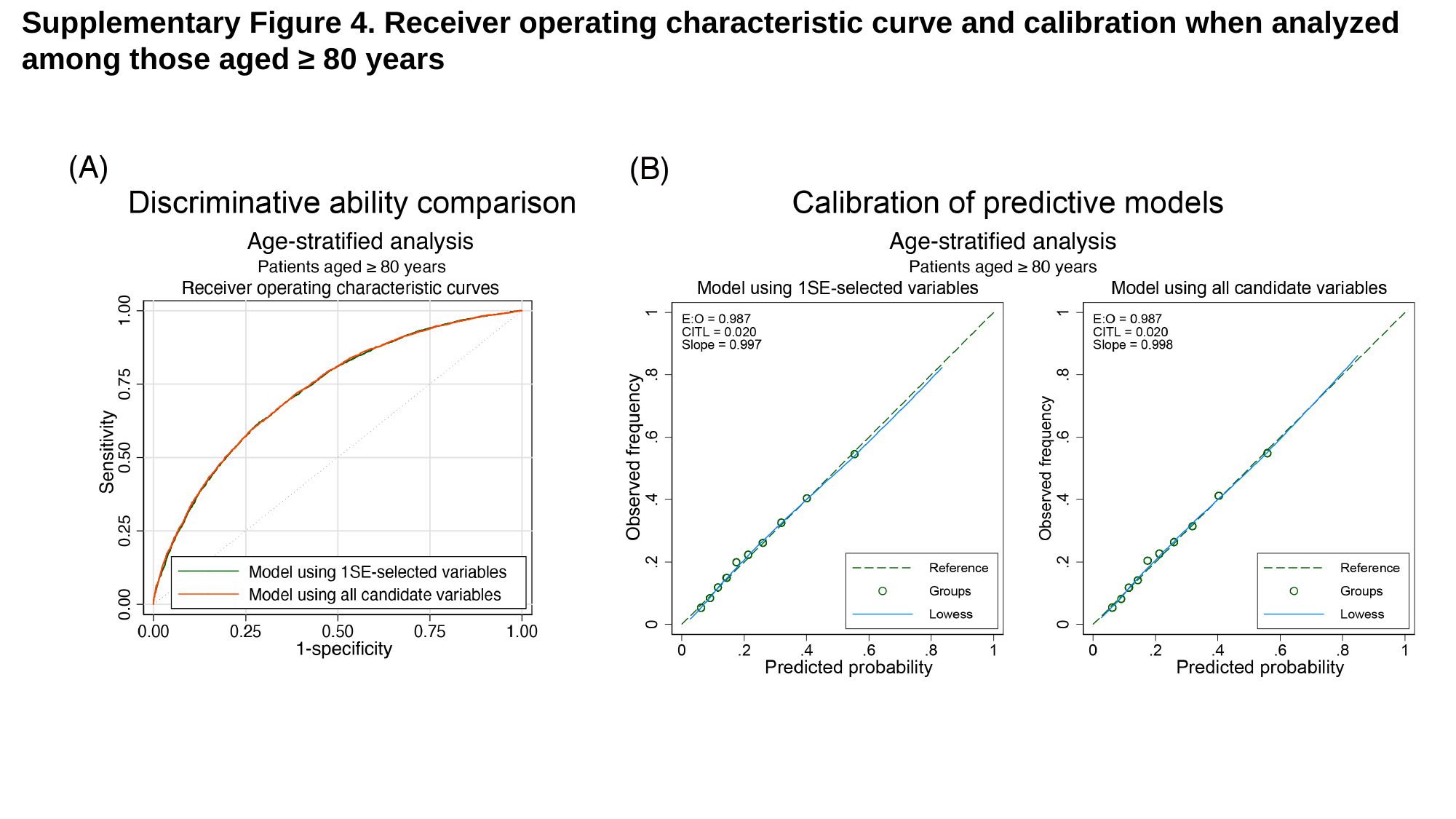

Supplementary Figure 4. Receiver operating characteristic curve and calibration when analyzed among those aged ≥ 80 years

## Slide 5
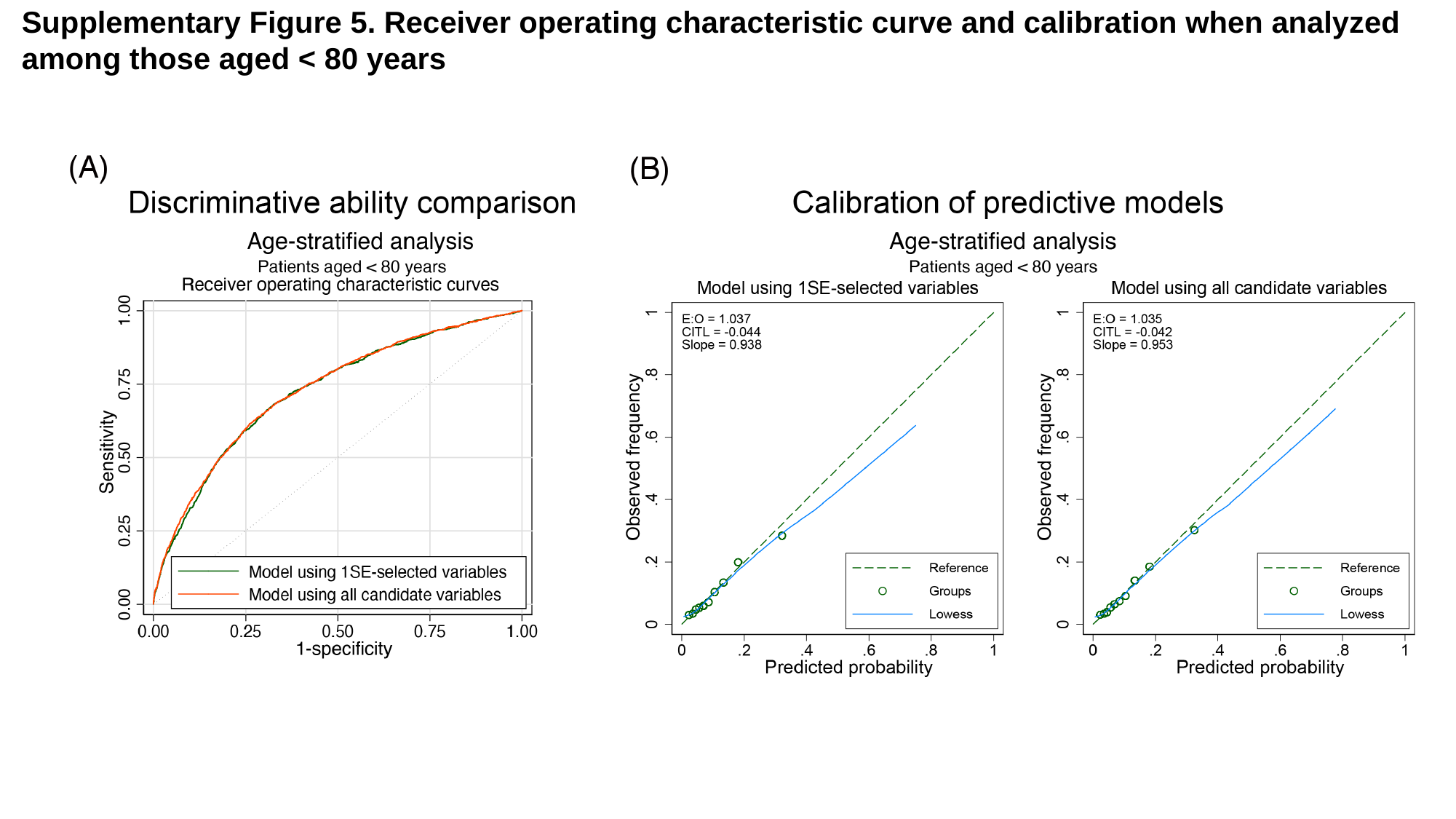

Supplementary Figure 5. Receiver operating characteristic curve and calibration when analyzed among those aged < 80 years

## Slide 6
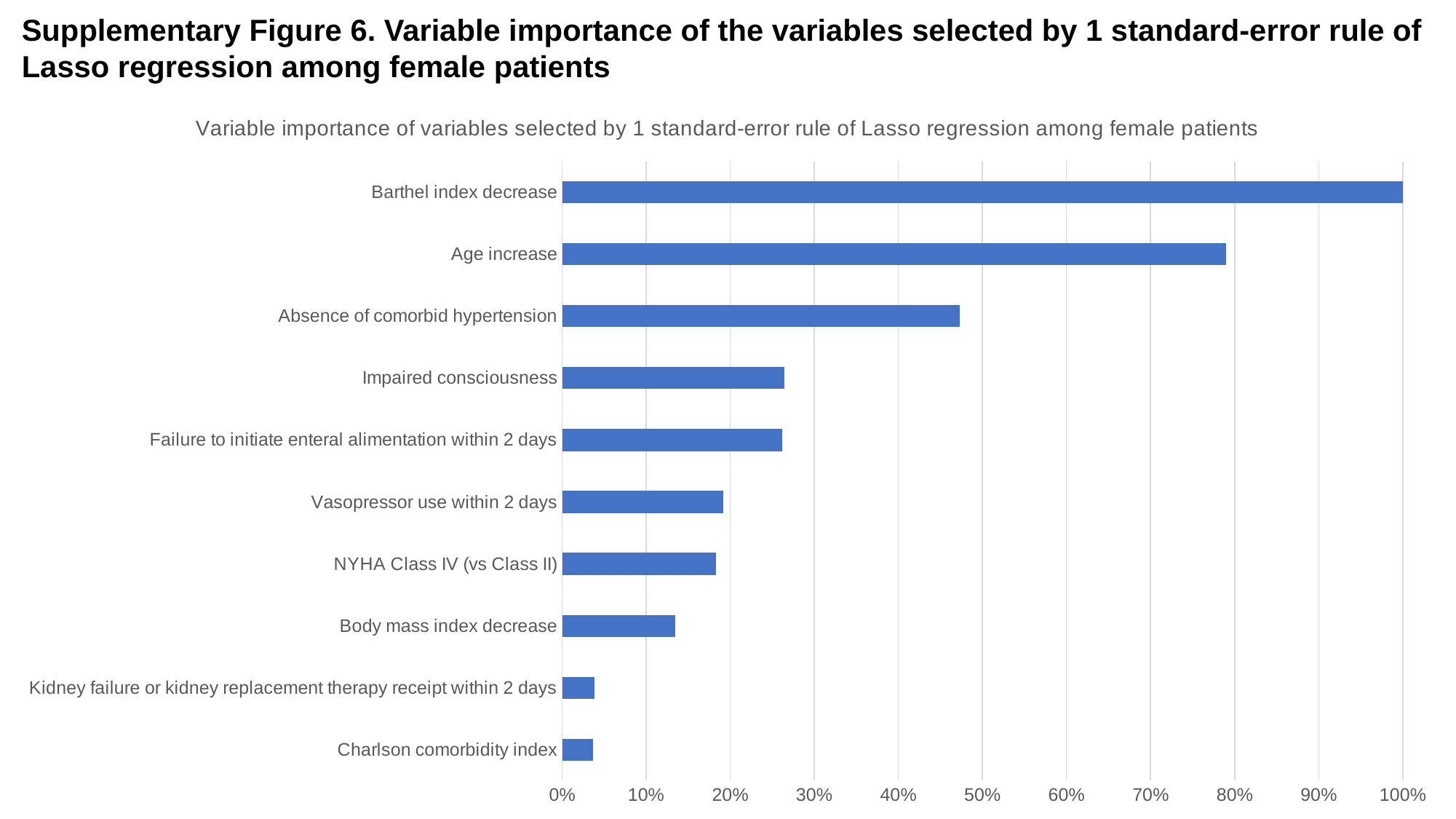

Supplementary Figure 6. Variable importance of the variables selected by 1 standard-error rule of Lasso regression among female patients
### Chart: Variable importance of variables selected by 1 standard-error rule of Lasso regression among female patients
| Category | |
|---|---|
| Charlson comorbidity index | 0.03705723067247513 |
| Kidney failure or kidney replacement therapy receipt within 2 days | 0.03845644858853829 |
| Body mass index decrease | 0.13446271060347553 |
| NYHA Class IV (vs Class II) | 0.18292723009680567 |
| Vasopressor use within 2 days | 0.19188835626384573 |
| Failure to initiate enteral alimentation within 2 days | 0.2622393185688377 |
| Impaired consciousness | 0.26453459624380976 |
| Absence of comorbid hypertension | 0.4729560779592893 |
| Age increase | 0.7902614702136233 |
| Barthel index decrease | 1.0 |

## Slide 7
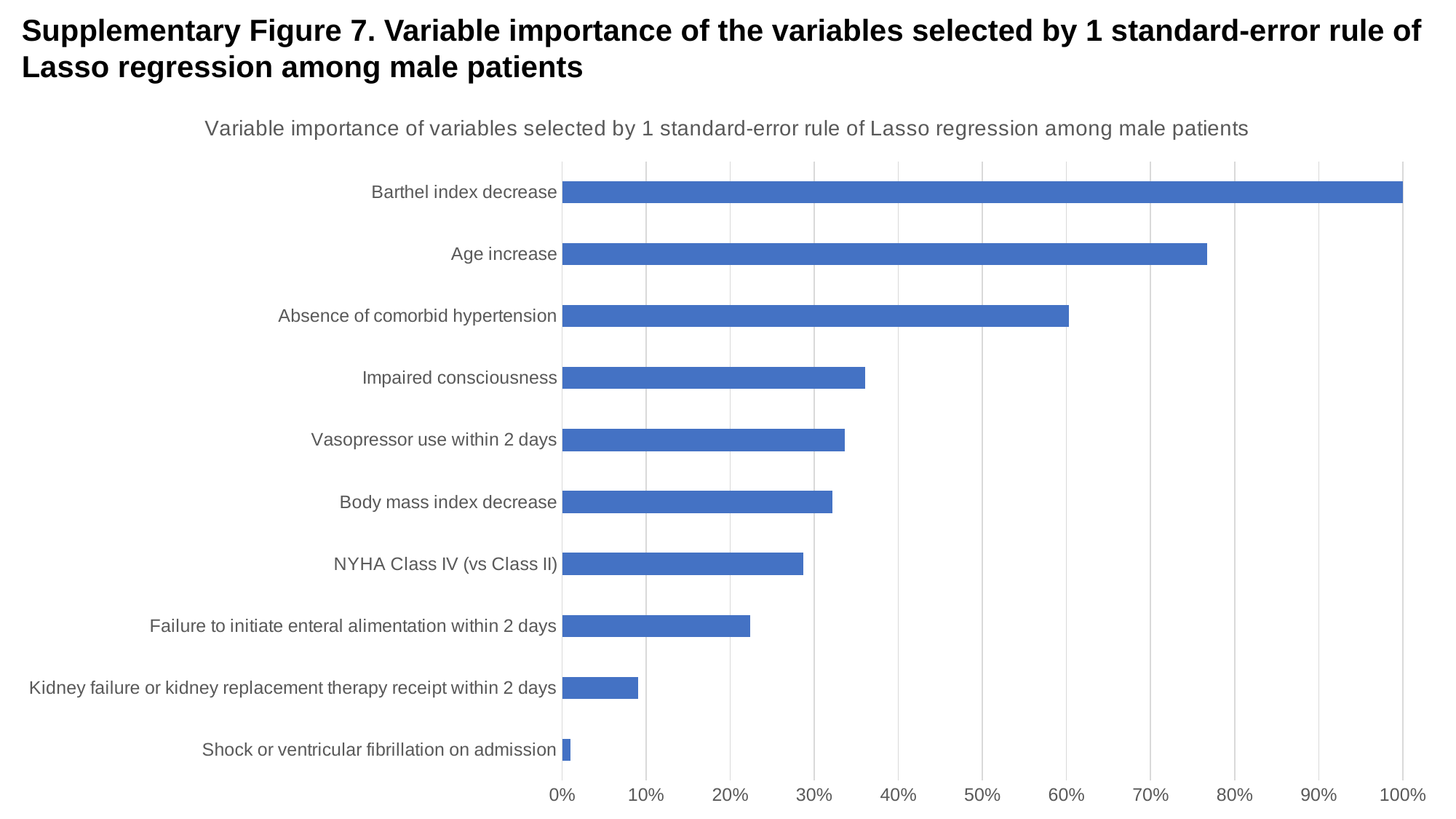

Supplementary Figure 7. Variable importance of the variables selected by 1 standard-error rule of Lasso regression among male patients
### Chart: Variable importance of variables selected by 1 standard-error rule of Lasso regression among male patients
| Category | |
|---|---|
| Shock or ventricular fibrillation on admission | 0.009783504486139587 |
| Kidney failure or kidney replacement therapy receipt within 2 days | 0.09060944573129022 |
| Failure to initiate enteral alimentation within 2 days | 0.2235352839103177 |
| NYHA Class IV (vs Class II) | 0.28696295176926745 |
| Body mass index decrease | 0.32200494435145266 |
| Vasopressor use within 2 days | 0.3363175691248898 |
| Impaired consciousness | 0.3603017528451602 |
| Absence of comorbid hypertension | 0.6032491194879441 |
| Age increase | 0.767367898469336 |
| Barthel index decrease | 1.0 |

## Slide 8
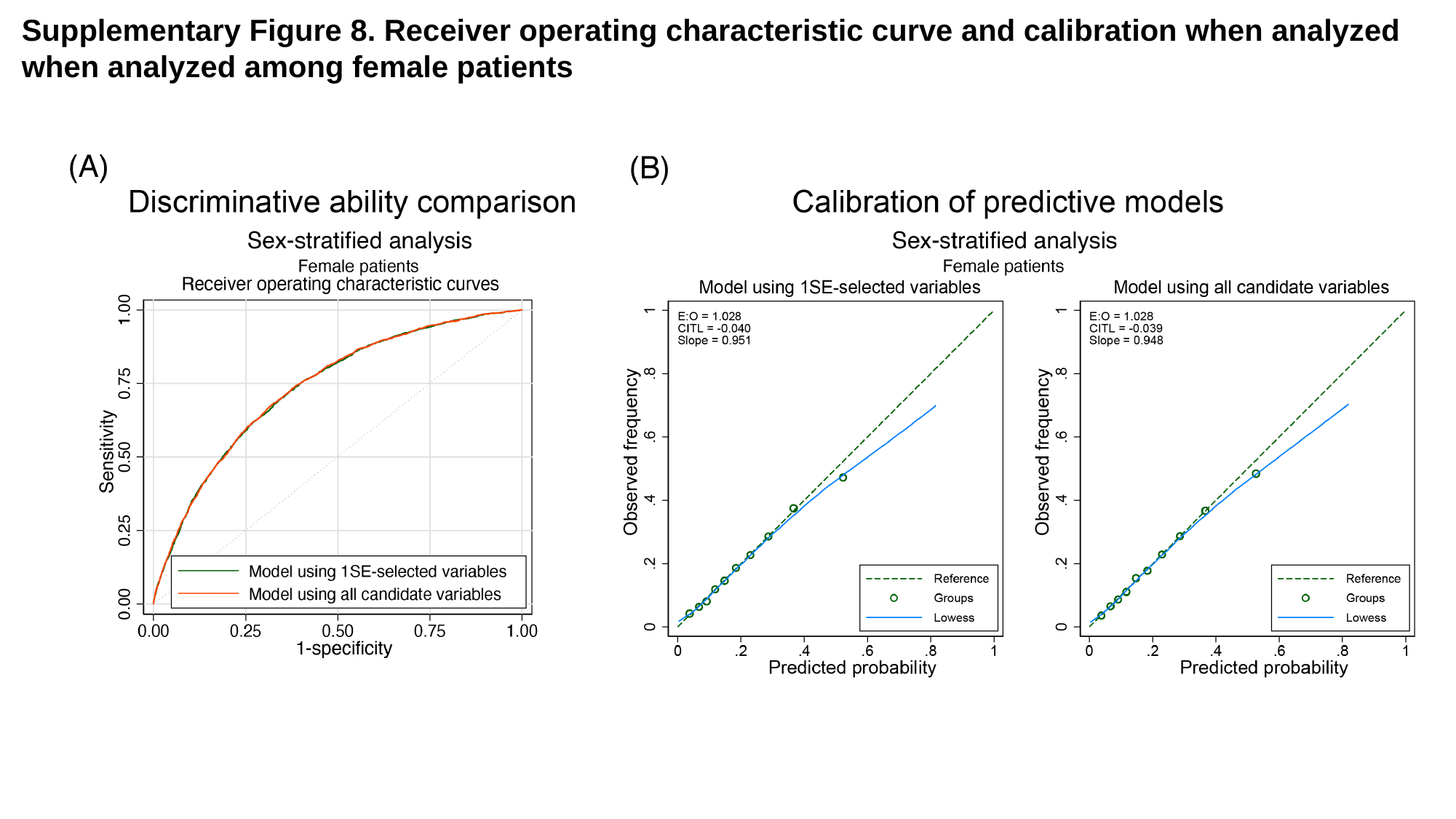

Supplementary Figure 8. Receiver operating characteristic curve and calibration when analyzed when analyzed among female patients

## Slide 9
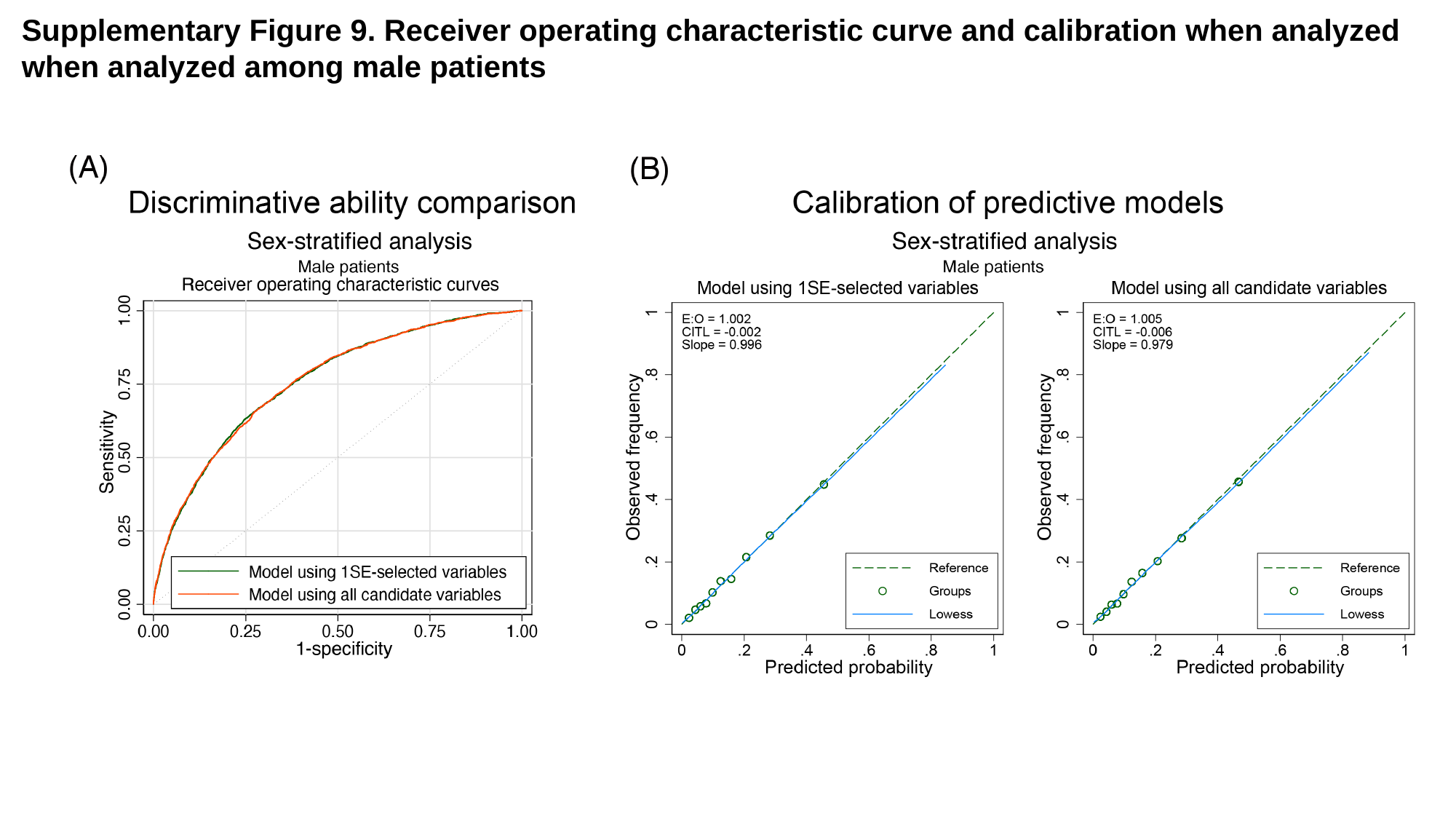

Supplementary Figure 9. Receiver operating characteristic curve and calibration when analyzed when analyzed among male patients

## Slide 10
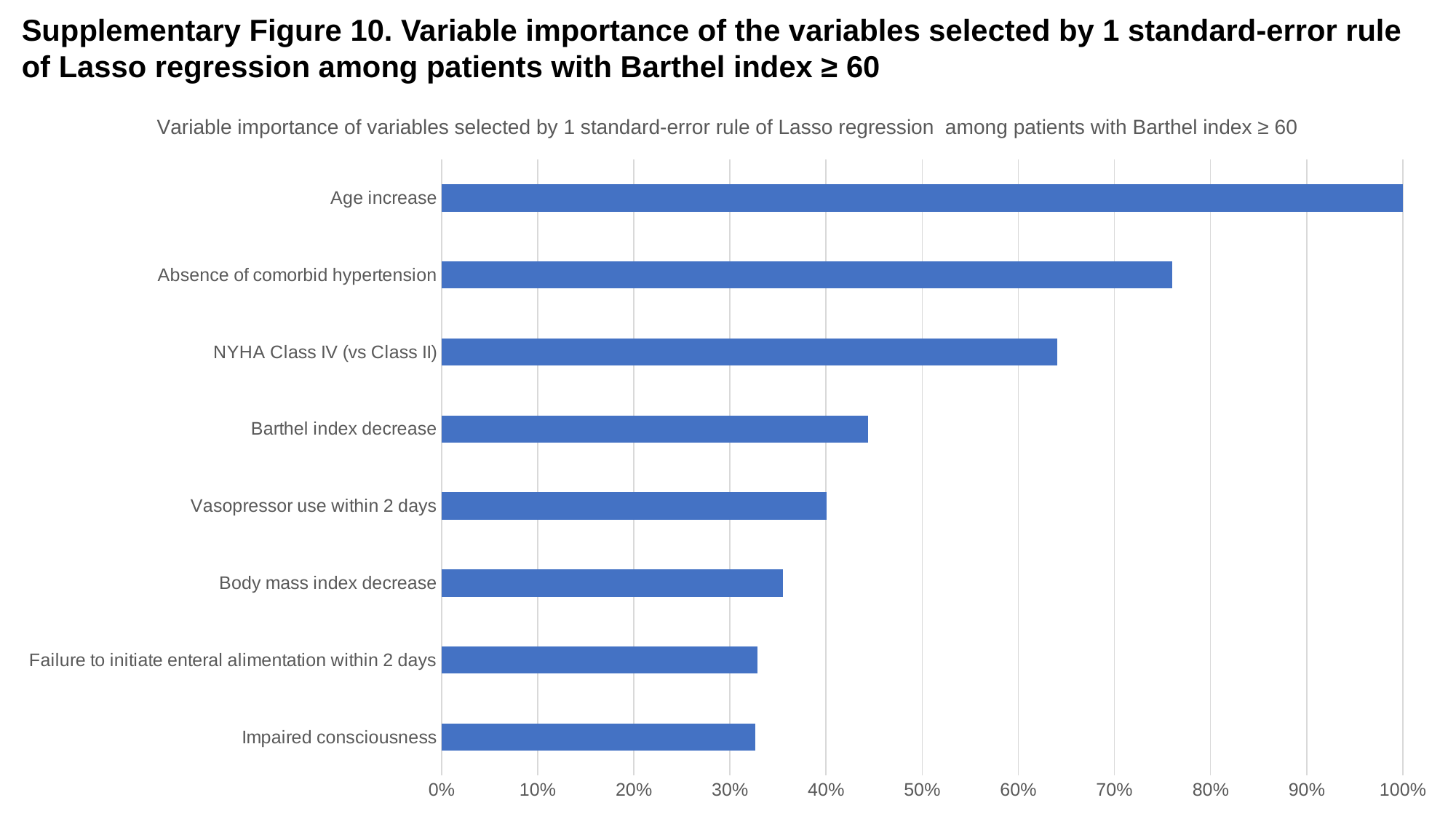

Supplementary Figure 10. Variable importance of the variables selected by 1 standard-error rule of Lasso regression among patients with Barthel index ≥ 60
### Chart: Variable importance of variables selected by 1 standard-error rule of Lasso regression among patients with Barthel index ≥ 60
| Category | |
|---|---|
| Impaired consciousness | 0.3261693423667231 |
| Failure to initiate enteral alimentation within 2 days | 0.32867663562040567 |
| Body mass index decrease | 0.3555192765312912 |
| Vasopressor use within 2 days | 0.4005532634275706 |
| Barthel index decrease | 0.44405357471885765 |
| NYHA Class IV (vs Class II) | 0.6401980675220078 |
| Absence of comorbid hypertension | 0.7600663116097047 |
| Age increase | 1.0 |

## Slide 11
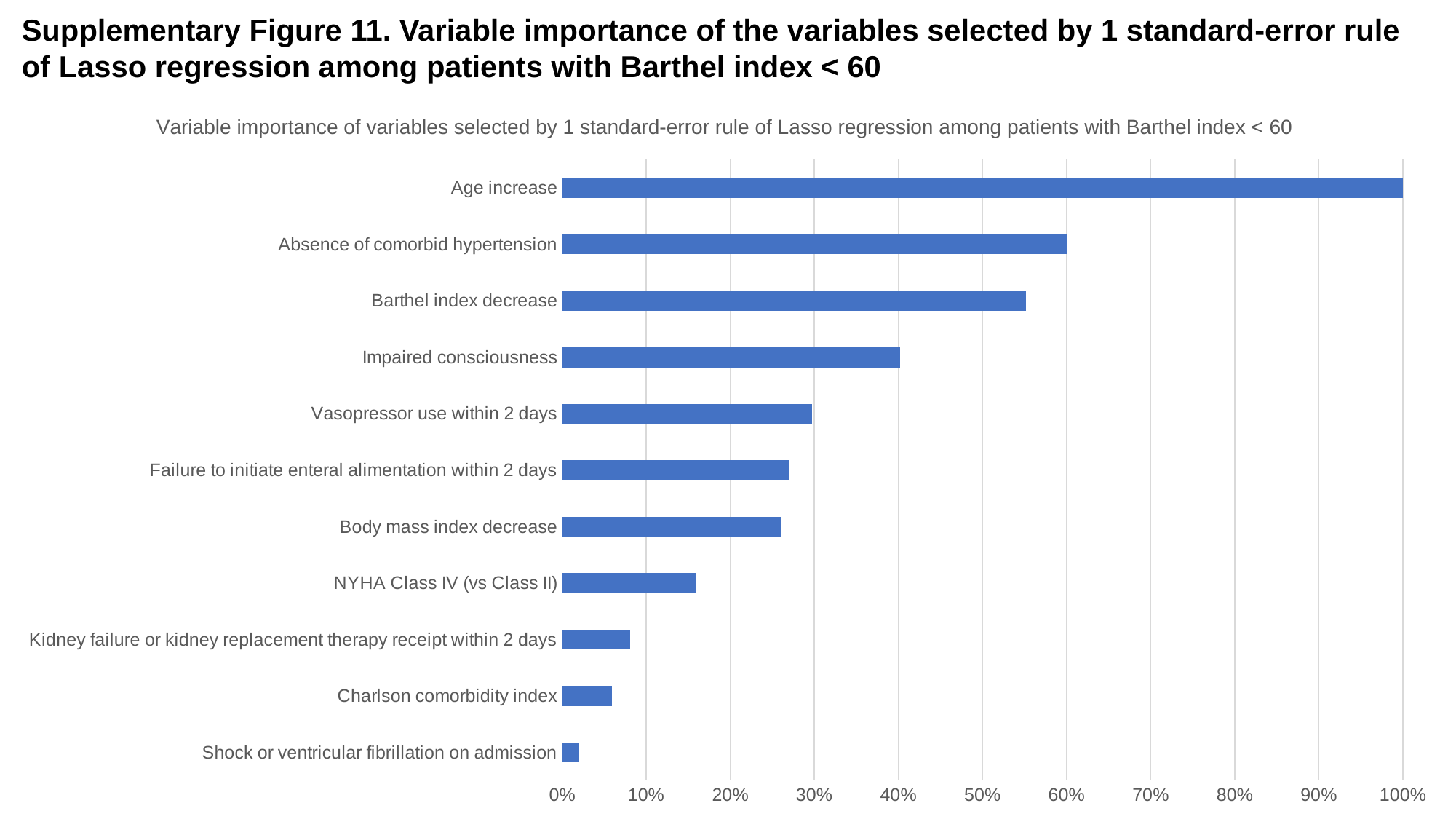

Supplementary Figure 11. Variable importance of the variables selected by 1 standard-error rule of Lasso regression among patients with Barthel index < 60
### Chart: Variable importance of variables selected by 1 standard-error rule of Lasso regression among patients with Barthel index < 60
| Category | |
|---|---|
| Shock or ventricular fibrillation on admission | 0.020742886179967248 |
| Charlson comorbidity index | 0.05939665340900664 |
| Kidney failure or kidney replacement therapy receipt within 2 days | 0.08081366733853043 |
| NYHA Class IV (vs Class II) | 0.15873396320019684 |
| Body mass index decrease | 0.26079940901529197 |
| Failure to initiate enteral alimentation within 2 days | 0.27046976879691903 |
| Vasopressor use within 2 days | 0.2970387169642183 |
| Impaired consciousness | 0.40198607283747506 |
| Barthel index decrease | 0.5521995059438732 |
| Absence of comorbid hypertension | 0.6015375174816483 |
| Age increase | 1.0 |

## Slide 12
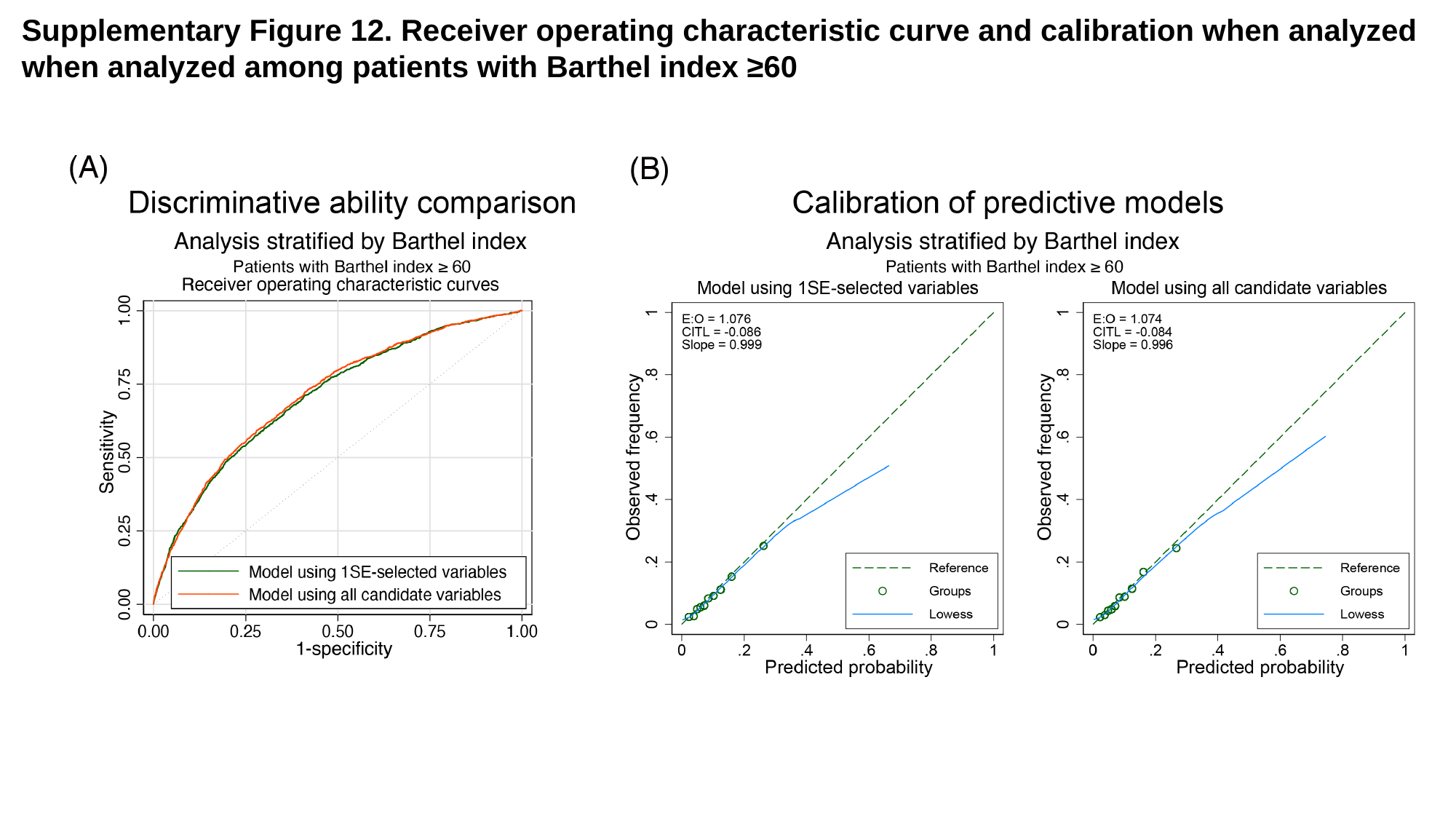

Supplementary Figure 12. Receiver operating characteristic curve and calibration when analyzed when analyzed among patients with Barthel index ≥60

## Slide 13
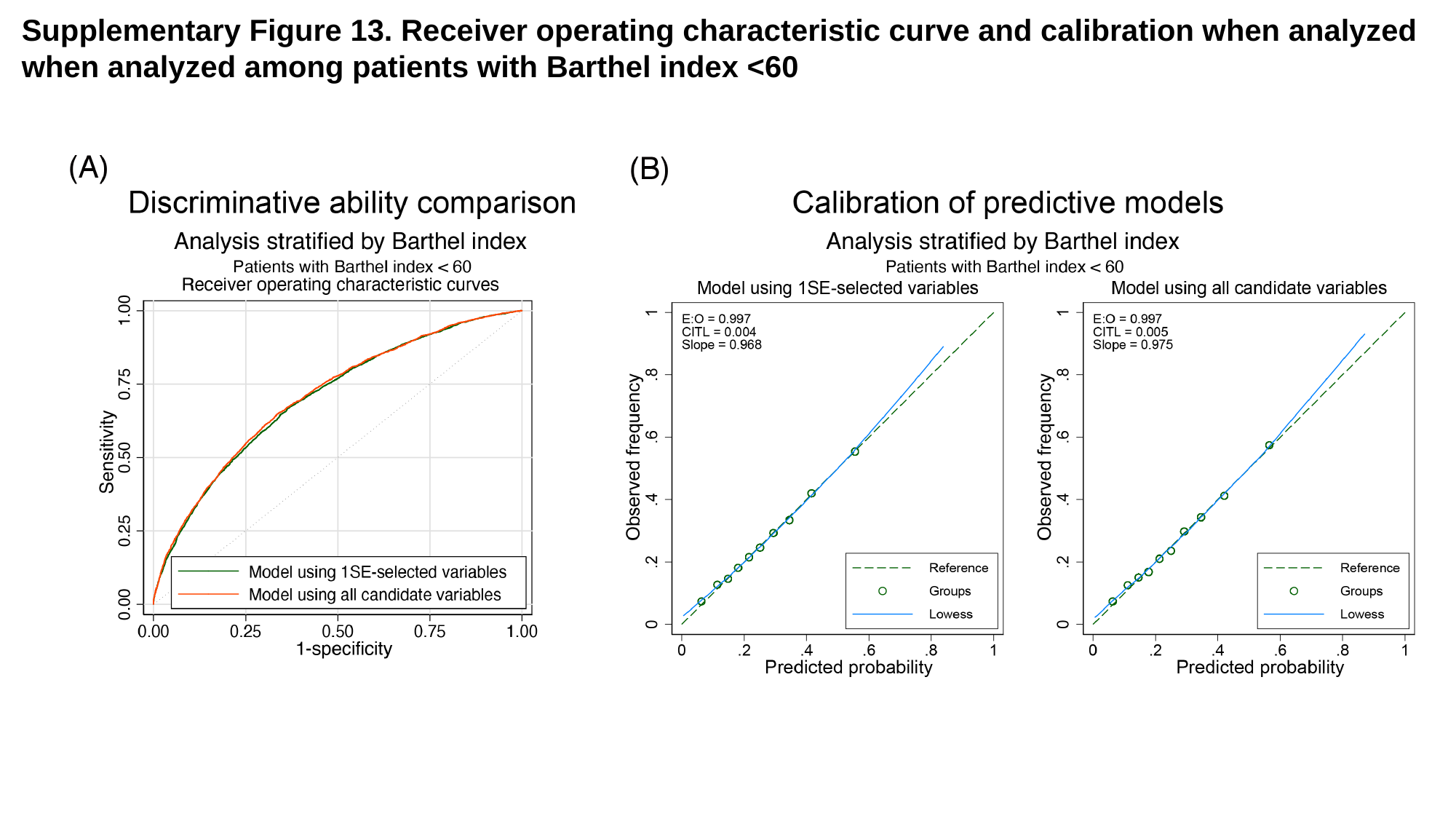

Supplementary Figure 13. Receiver operating characteristic curve and calibration when analyzed when analyzed among patients with Barthel index <60

## Slide 14
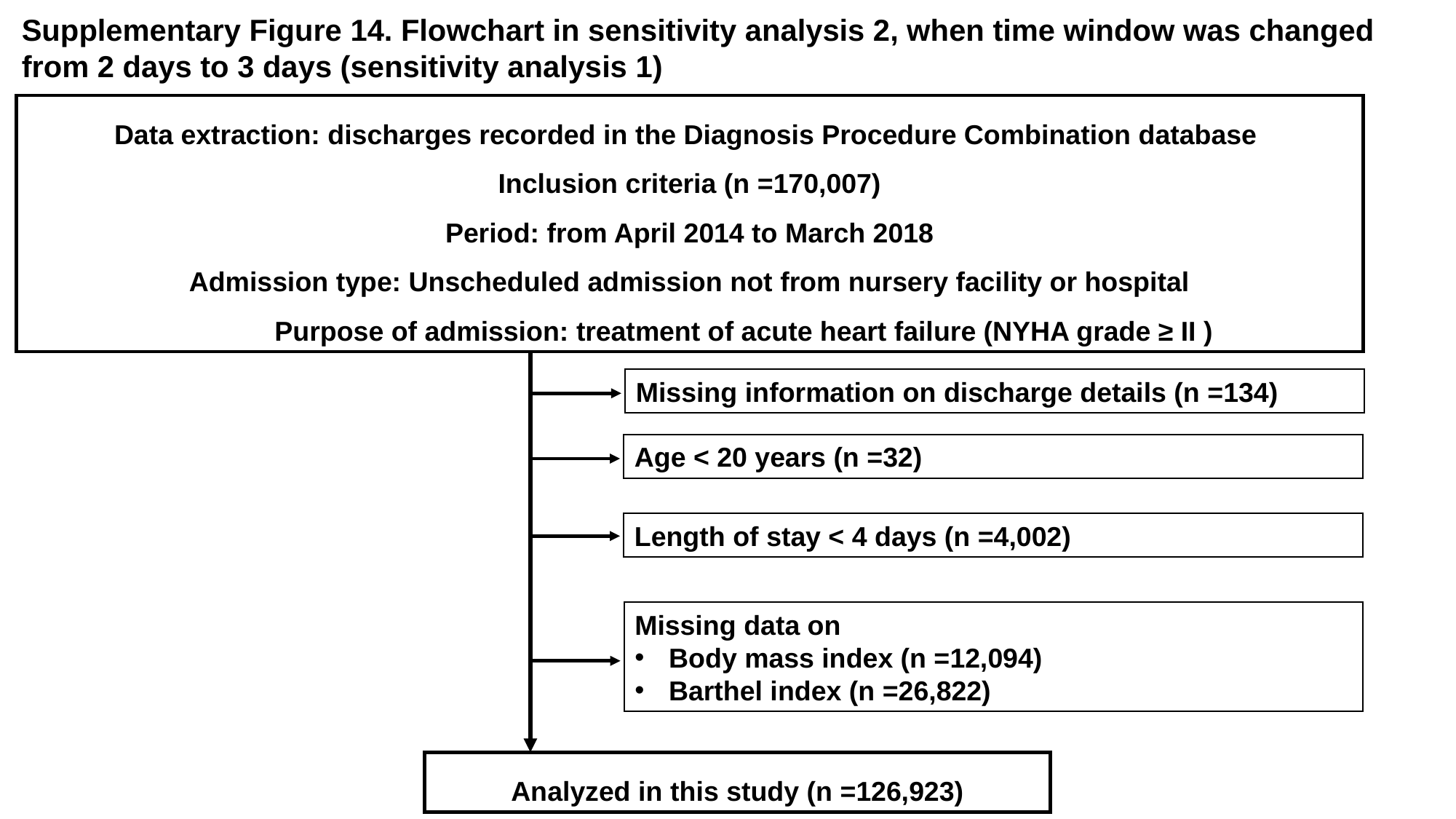

Supplementary Figure 14. Flowchart in sensitivity analysis 2, when time window was changed from 2 days to 3 days (sensitivity analysis 1)
Data extraction: discharges recorded in the Diagnosis Procedure Combination database
Inclusion criteria (n =170,007)
Period: from April 2014 to March 2018
Admission type: Unscheduled admission not from nursery facility or hospital
 	Purpose of admission: treatment of acute heart failure (NYHA grade ≥ II )
Missing information on discharge details (n =134)
Age < 20 years (n =32)
Length of stay < 4 days (n =4,002)
Missing data on
Body mass index (n =12,094)
Barthel index (n =26,822)
Analyzed in this study (n =126,923)

## Slide 15
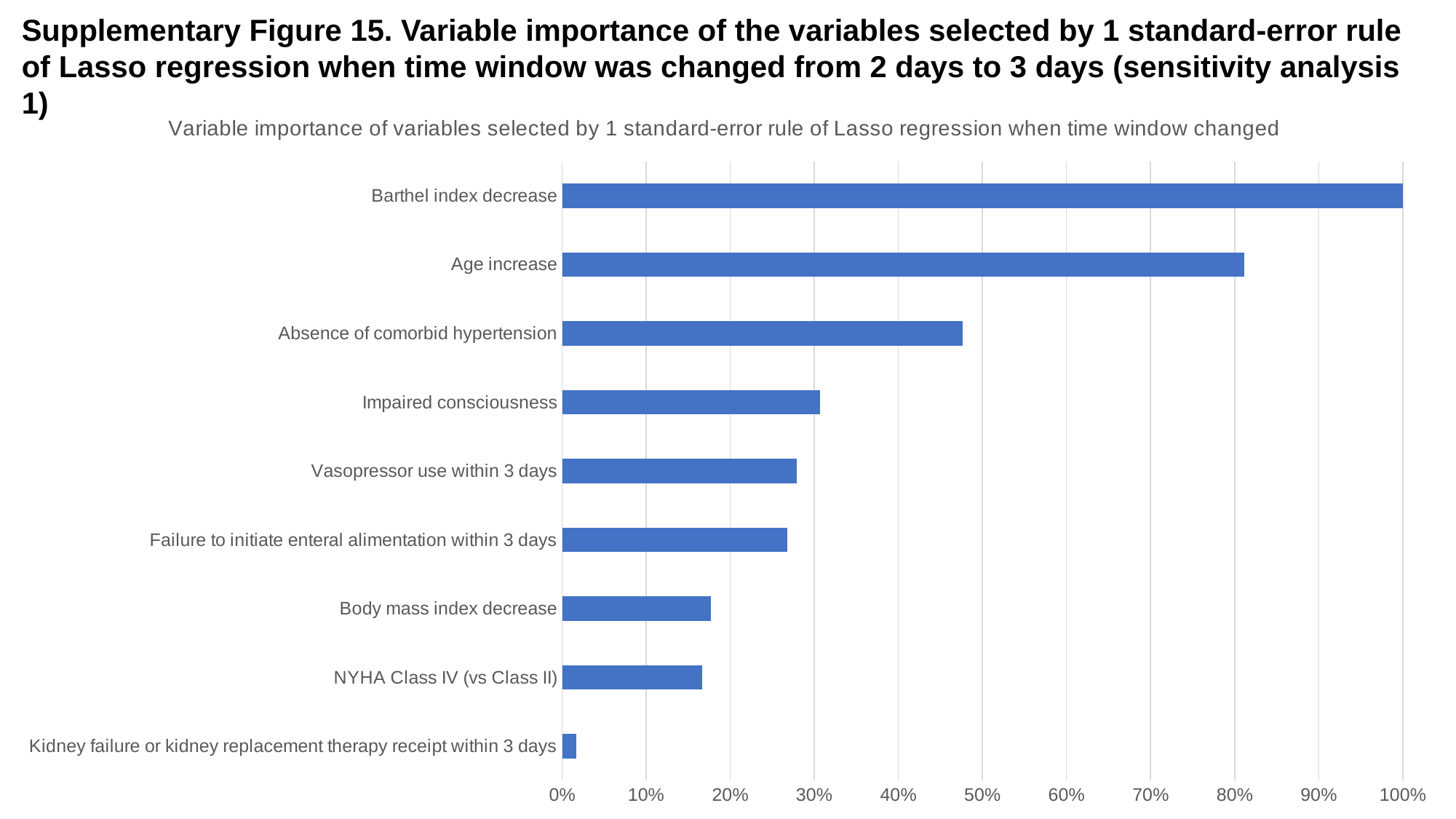

Supplementary Figure 15. Variable importance of the variables selected by 1 standard-error rule of Lasso regression when time window was changed from 2 days to 3 days (sensitivity analysis 1)
### Chart: Variable importance of variables selected by 1 standard-error rule of Lasso regression when time window changed
| Category | |
|---|---|
| Kidney failure or kidney replacement therapy receipt within 3 days | 0.016559495183933726 |
| NYHA Class IV (vs Class II) | 0.16692656205149045 |
| Body mass index decrease | 0.1773350111892247 |
| Failure to initiate enteral alimentation within 3 days | 0.2678241068571592 |
| Vasopressor use within 3 days | 0.27931005565857064 |
| Impaired consciousness | 0.3071347806909425 |
| Absence of comorbid hypertension | 0.47612313442194665 |
| Age increase | 0.8114461221032855 |
| Barthel index decrease | 1.0 |

## Slide 16
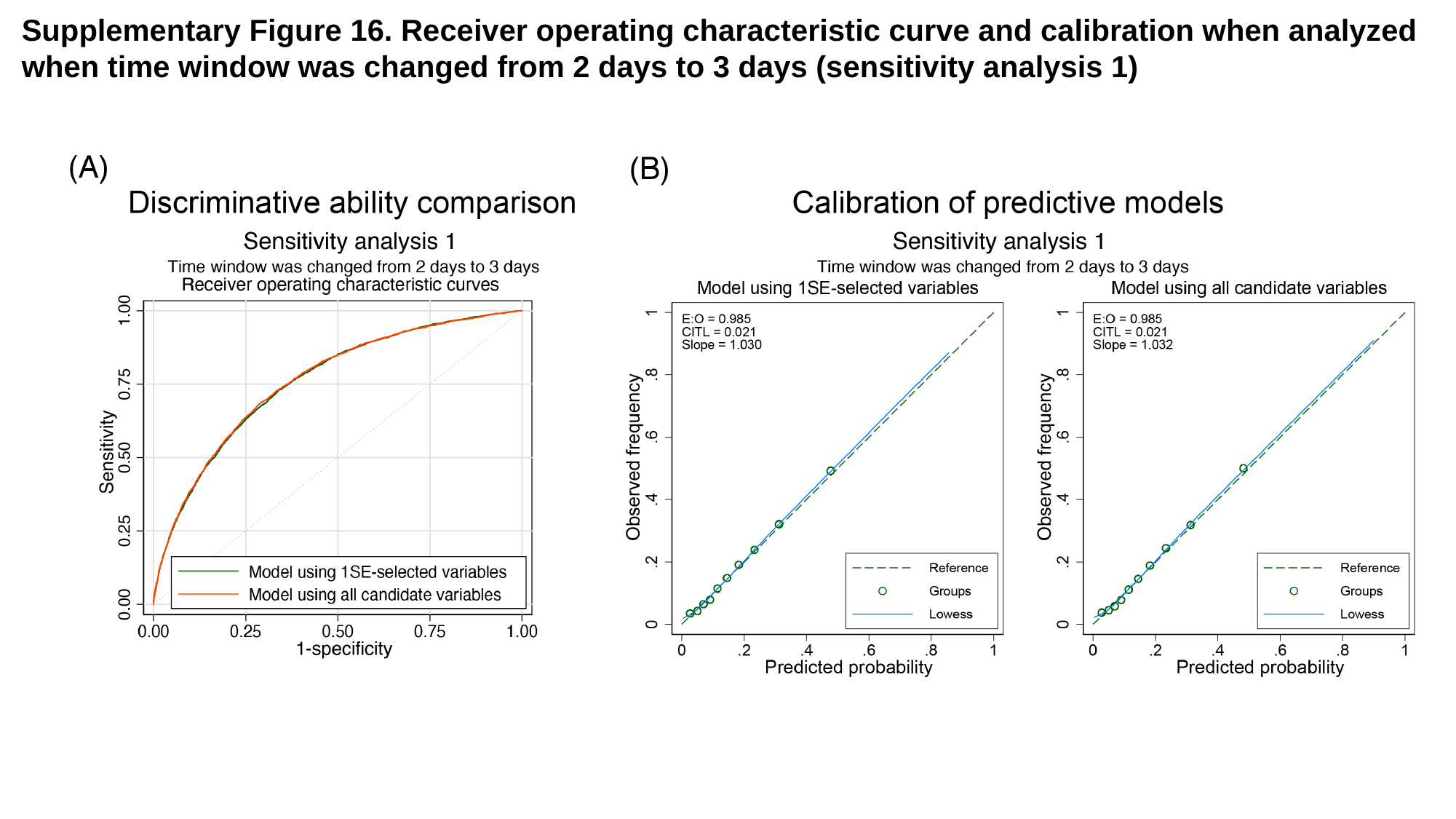

Supplementary Figure 16. Receiver operating characteristic curve and calibration when analyzed when time window was changed from 2 days to 3 days (sensitivity analysis 1)

## Slide 17
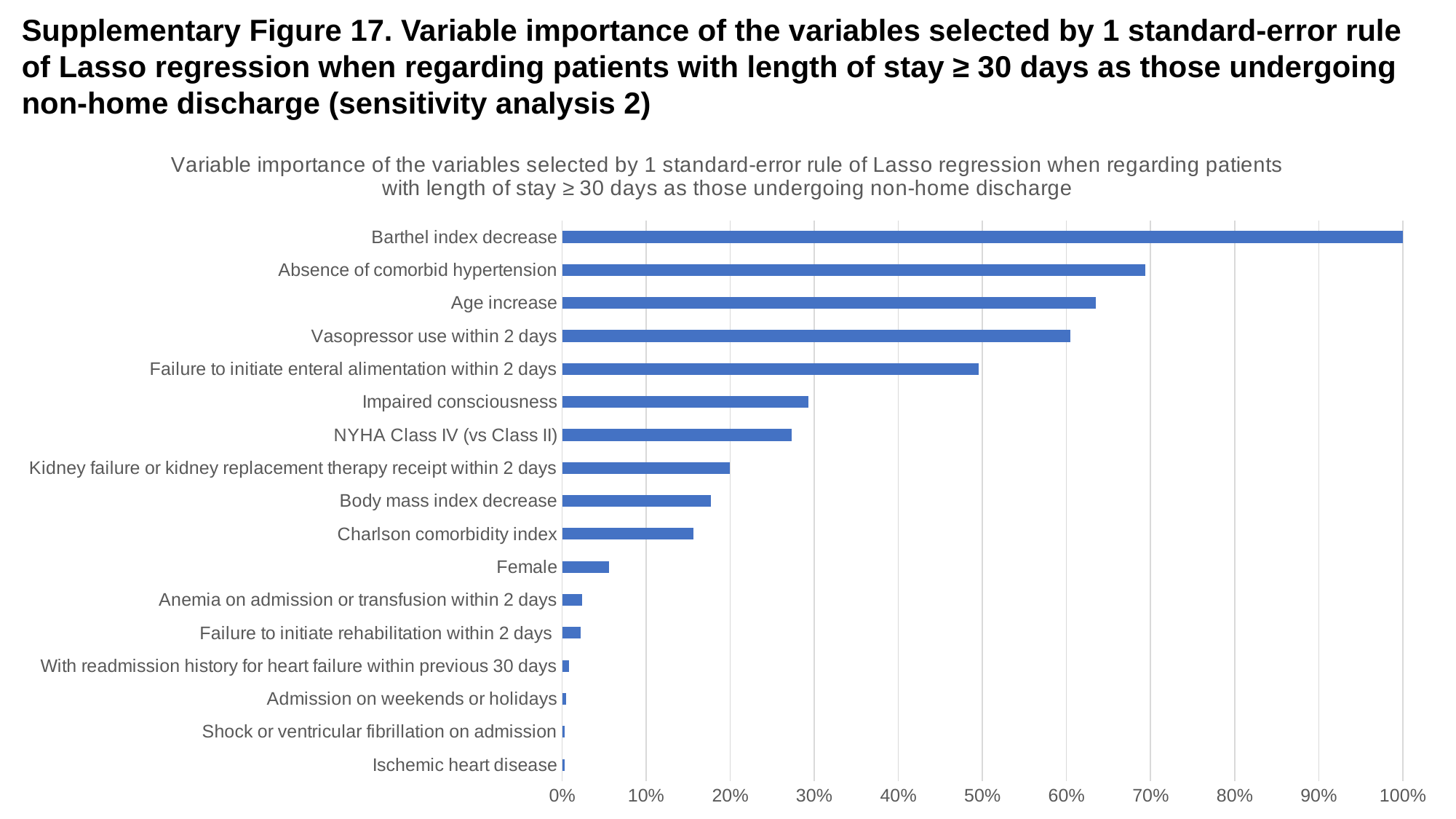

Supplementary Figure 17. Variable importance of the variables selected by 1 standard-error rule of Lasso regression when regarding patients with length of stay ≥ 30 days as those undergoing non-home discharge (sensitivity analysis 2)
### Chart: Variable importance of the variables selected by 1 standard-error rule of Lasso regression when regarding patients with length of stay ≥ 30 days as those undergoing non-home discharge
| Category | |
|---|---|
| Ischemic heart disease | 0.003143043080585186 |
| Shock or ventricular fibrillation on admission | 0.003369686647881296 |
| Admission on weekends or holidays | 0.004650761456588569 |
| With readmission history for heart failure within previous 30 days | 0.008291411262242051 |
| Failure to initiate rehabilitation within 2 days | 0.0220845201182133 |
| Anemia on admission or transfusion within 2 days | 0.023589511134802774 |
| Female | 0.05620228633857828 |
| Charlson comorbidity index | 0.15654408924792756 |
| Body mass index decrease | 0.17690747511599883 |
| Kidney failure or kidney replacement therapy receipt within 2 days | 0.19922992325108332 |
| NYHA Class IV (vs Class II) | 0.27344832767014193 |
| Impaired consciousness | 0.2931314419163981 |
| Failure to initiate enteral alimentation within 2 days | 0.4957661324758242 |
| Vasopressor use within 2 days | 0.6043753458845732 |
| Age increase | 0.635206098539291 |
| Absence of comorbid hypertension | 0.6937489024509108 |
| Barthel index decrease | 1.0 |

## Slide 18
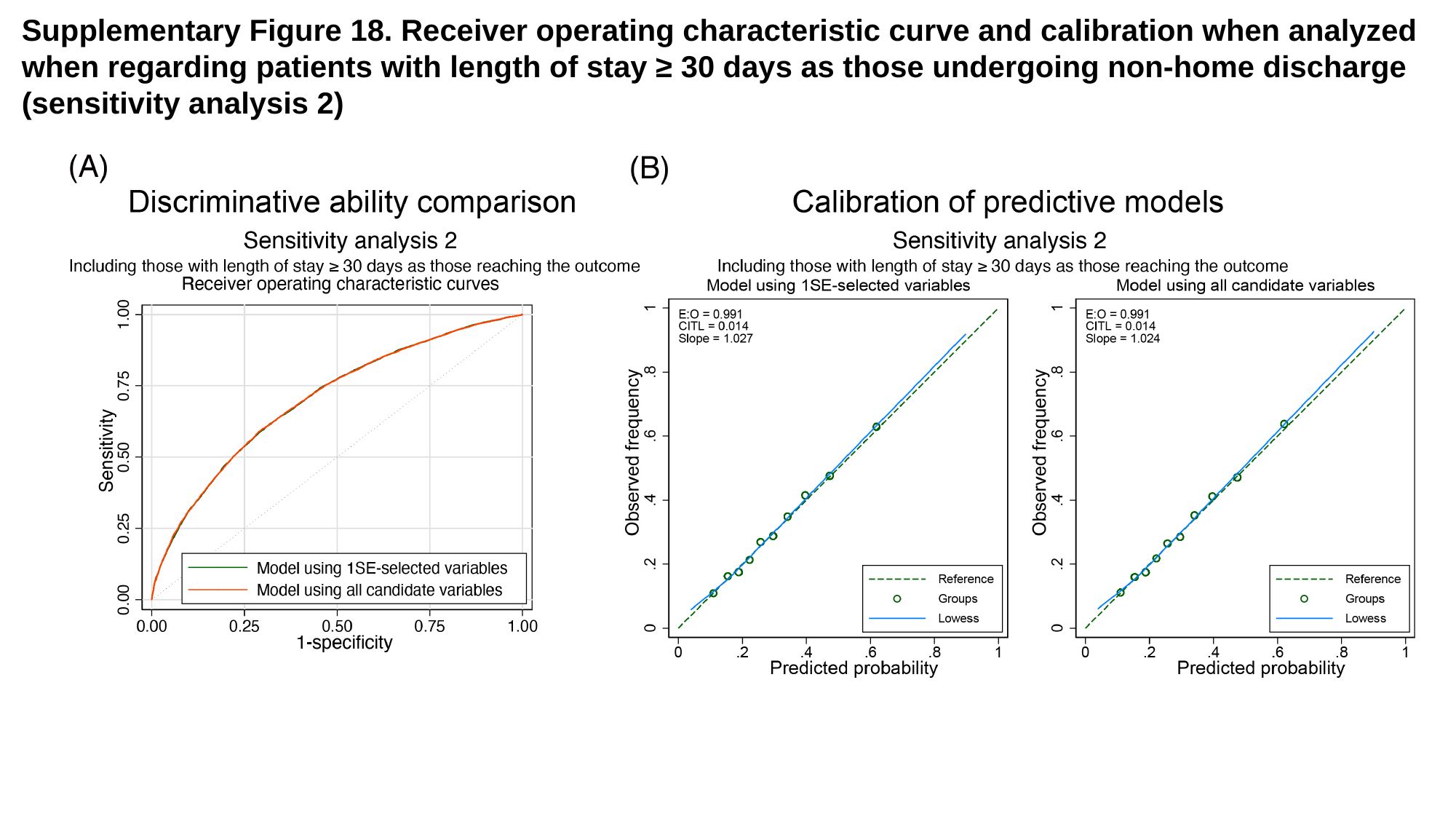

Supplementary Figure 18. Receiver operating characteristic curve and calibration when analyzed when regarding patients with length of stay ≥ 30 days as those undergoing non-home discharge (sensitivity analysis 2)

## Slide 19
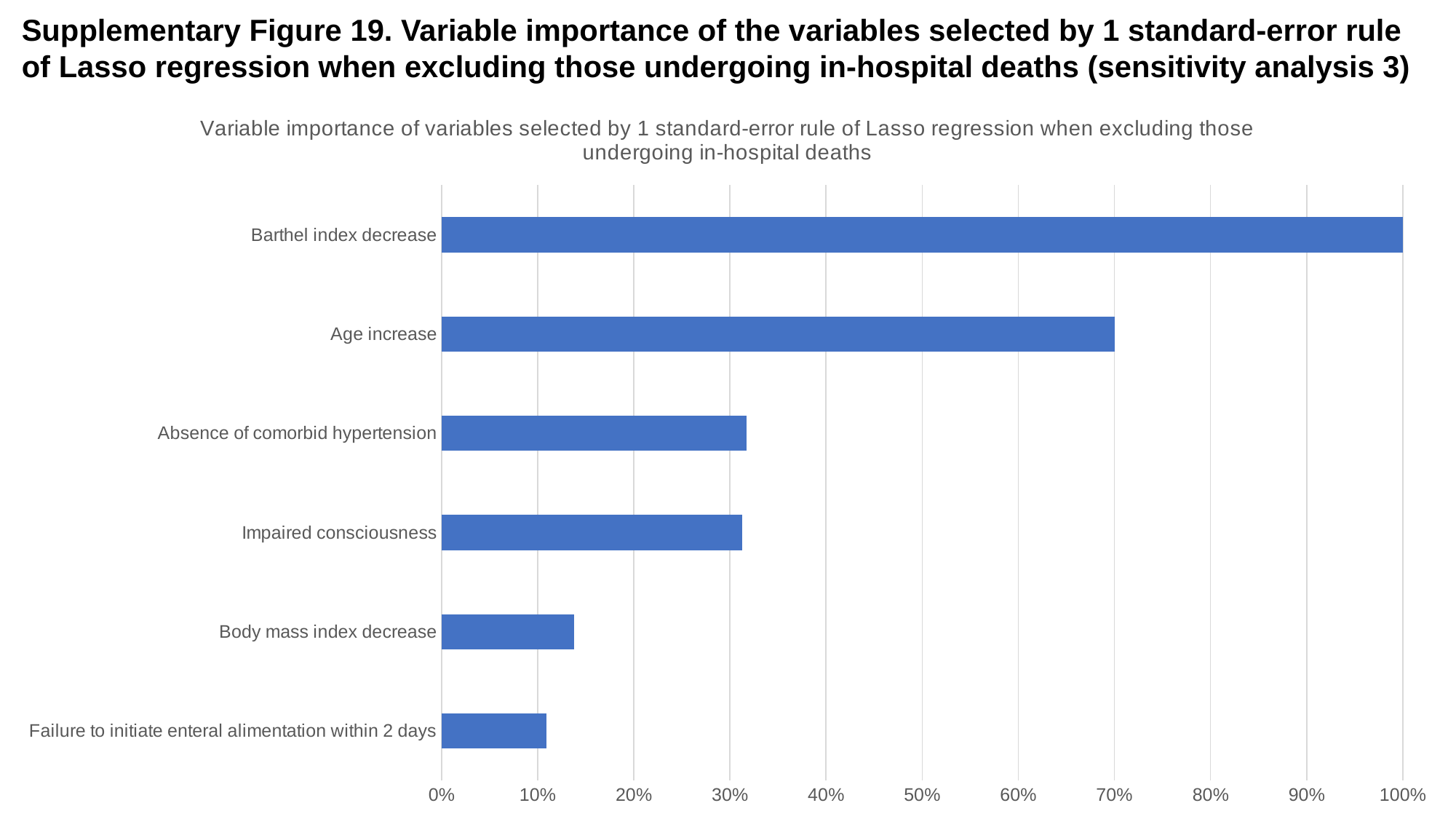

Supplementary Figure 19. Variable importance of the variables selected by 1 standard-error rule of Lasso regression when excluding those undergoing in-hospital deaths (sensitivity analysis 3)
### Chart: Variable importance of variables selected by 1 standard-error rule of Lasso regression when excluding those undergoing in-hospital deaths
| Category | |
|---|---|
| Failure to initiate enteral alimentation within 2 days | 0.1091447930853579 |
| Body mass index decrease | 0.13798041896451937 |
| Impaired consciousness | 0.3130286040190882 |
| Absence of comorbid hypertension | 0.3171073769804255 |
| Age increase | 0.7006095442196124 |
| Barthel index decrease | 1.0 |

## Slide 20
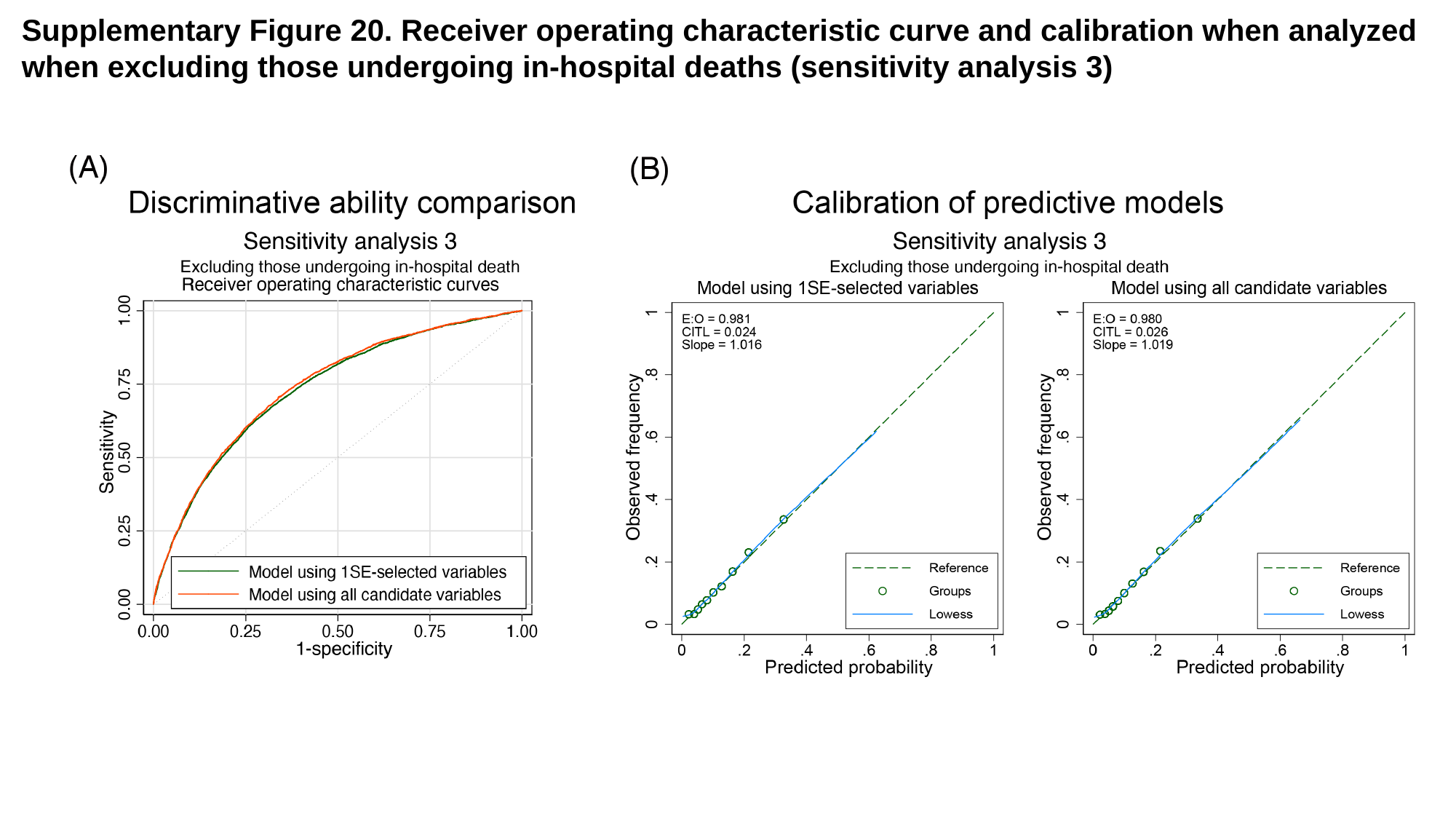

Supplementary Figure 20. Receiver operating characteristic curve and calibration when analyzed when excluding those undergoing in-hospital deaths (sensitivity analysis 3)
